# Stroma-oriented tumor microenvironment facilitates intraosseous ameloblastoma tumor growth

**DOI:** 10.1016/j.isci.2026.117344

**Published:** 2026-08-27

**Authors:** Da-Yo Yuh, Chi-Ju Yao, Chia-Yu Chen, Ching-Yi Wu, Jian-Liang Chou, Hui-Na Chou, Ling-Yu Kung, Yi-Tzu Chen, Wei-Chin Chang, Yuan-Wu Chen, Qunzhou Zhang, Anh D. Le, Ting-Han Chang

**Affiliations:** 1 Institute of Oral Biology, College of Dentistry, National Yang Ming Chiao Tung University, Taipei, Taiwan; 2 School of Dentistry, College of Oral Medicine, National Defense Medical University, Taipei, Taiwan; 3 Division of Family Dentistry and Oral Diagnosis, Department of Dentistry, Tri-Service General Hospital, Taipei, Taiwan; 4 Department of Dentistry, College of Dentistry, National Yang Ming Chiao Tung University, Taipei, Taiwan; 5 Department of Stomatology, Taipei Veterans General Hospital, Taipei, Taiwan; 6 Instrument Center, National Defense Medical University, Taipei, Taiwan; 7 School of Dentistry, Chung Shan Medical University, Taichung, Taiwan; 8 Department of Oral and Maxillofacial Surgery, Chung Shan Medical University Hospital, Taichung, Taiwan; 9 Division of Oral and Maxillofacial Surgery, Department of Dentistry, Tri-Service General Hospital, Taipei, Taiwan; 10 Department of Oral and Maxillofacial Surgery and Pharmacology, University of Pennsylvania School of Dental Medicine, Philadelphia, PA, USA; 11 Department of Oral & Maxillofacial Surgery, University of Pennsylvania Health System, Perelman Center for Advanced Medicine, Philadelphia, PA, USA

**Keywords:** ameloblastoma, tumor microenvironment, mesenchymal stromal cells, secretome, miRNA, single-nucleus ATAC and RNA sequence, orthotopic mouse model

## Abstract

Ameloblastoma (AM) is a locally aggressive odontogenic epithelial tumor with poorly understood intraosseous pathogenesis. Here, we established an orthotopic intraosseous mouse model using AM epithelial cells in combination with AM-derived mesenchymal stromal cells (AMMSCs) or their secretome. AMMSC-derived extracellular vesicle-enriched secretome enhances stemness, epithelial-mesenchymal transition (EMT) phenotypes, and tumorigenic ability of AM epithelial cells. A unique signature of microRNAs in AMMSC-derived secretome is functionally associated with the upregulation of EMT-regulatory transcription factors in tumor epithelial cells. Single-nucleus assay for transposase-accessible chromatin (ATAC) and RNA sequencing revealed dynamic EMT states in primary human AM-derived epithelial cells treated with paired AMMSC-derived secretome. This study suggests that AMMSCs and their secretome drive orthotopic AM tumor development by promoting tumor epithelial cell proliferation, EMT, and vascular formation in the tumor microenvironment. This stromal-oriented orthotopic model provides a clinically relevant platform for dissecting tumor-stroma crosstalk and identifying therapeutic vulnerabilities in AM.

## Highlights

• AMMSC-derived EV-enriched secretome drives EMT via a distinct microRNA signature
• Stroma-oriented orthotopic intraosseous mouse model of human ameloblastoma
• Single-nucleus multi-omics reveals an EMT continuum in ameloblastoma epithelium
• AMMSCs orchestrate histological patterning and vascular-like structures *in vivo*

## Graphical abstract

**Figure:**
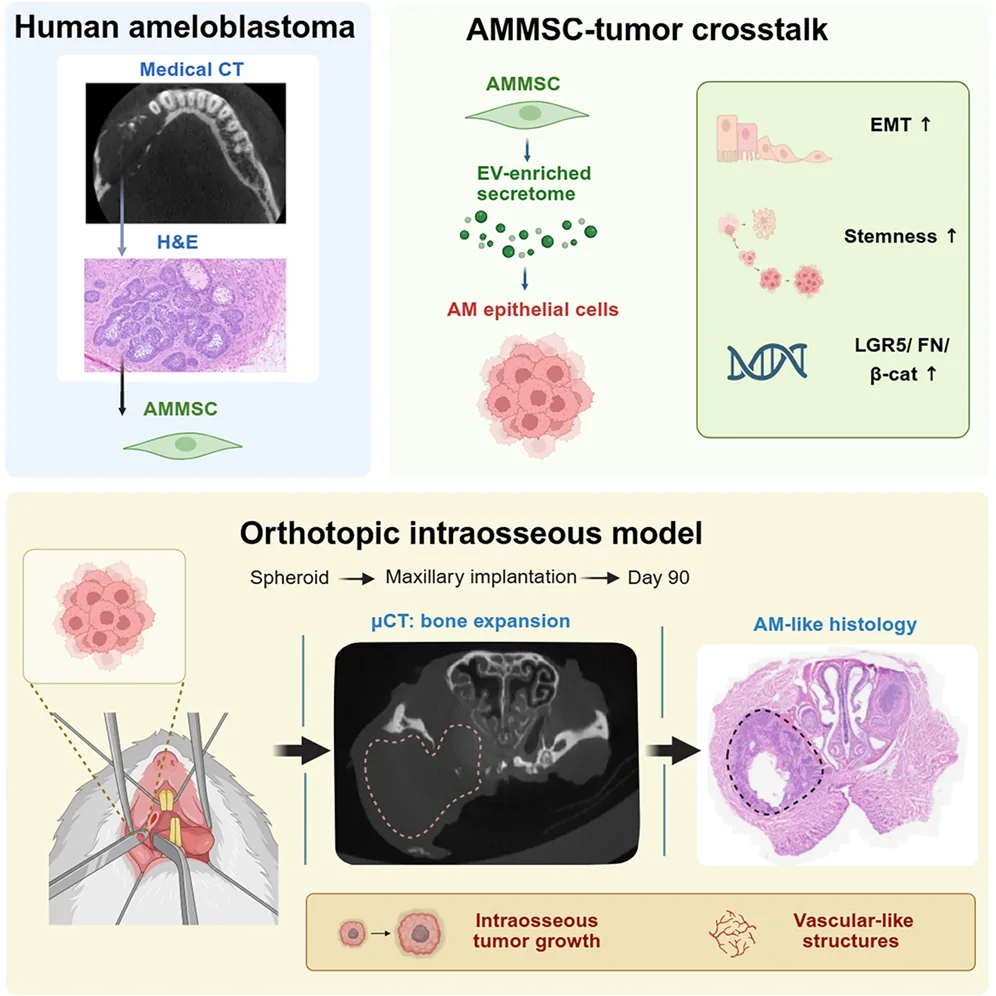

## Teaser

Immunology; Cell biology

## Introduction

Ameloblastoma (AM) is one of the most common odontogenic epithelial tumors of the jawbone, characterized by locally aggressive behavior and a high recurrence rate.^1^^,^^2^ Although several targetable single-nucleotide variants (SNVs) in tumor epithelial cells have been identified in AM,^3^ there is still a lack of experimental models that faithfully recapitulates the intraosseous tumor microenvironment for studying drug pharmacokinetics and therapeutic efficacy.

Conventional AM has histopathological subtypes characterized by odontogenic epithelium tumor cells within varying degrees of collagenization in the surrounding stroma.^4^^,^^5^ The tumor stroma provides extracellular matrix and soluble factors that drive tumor growth, invasion, angiogenesis, and therapeutic resistance.^6^^,^^7^^,^^8^ Recent studies demonstrated that IL-6 secreted by AM-derived mesenchymal stromal cells (AMMSCs) promotes epithelial-mesenchymal transition (EMT) and stemness in primary AM-derived epithelial cells (AMEpiCs).^9^^,^^10^ While prior studies have focused on exploring the role of AM epithelial cells in tumor progression, much less has been explored on the role of AMMSCs and their secretome in AM pathogenesis, progression, and drug resistance.

Currently, several animal models of human AM are available, including subcutaneous transplantation of AM tumor cells mixed with matrigel or hydrogel into the scalp^11^ or flank^9^ of nude mice, and transplantation of AM tumor cells mixed with hydroxyapatite (HA) and tricalcium phosphate (TCP) into the flank of nude mice.^12^ Patient-derived AM xenograft (PDX) models have also been reported, in which human AM tissues are transplanted subcutaneously into the flank of nude mice and tracked by labeling epidermal growth factor receptor (EGFR).^13^^,^^14^ However, the mystery of the odontogenic tumor development is the dysregulated crosstalk between the dental epithelium and the surrounding ectomesenchyme in the jawbone. Hence, existing subcutaneous animal models of AM could not mimic the intraosseous microenvironment, thus having impeded exploring the important role of pertinent stromal cells in the pathogenesis and progression of AM.^11^^,^^12^^,^^15^^,^^16^

In this study, we performed microRNA (miRNA) and mRNA sequences, and single-nucleus assay for transposase-accessible chromatin (ATAC) and RNA sequencing to identify the functional signature network of miRNAs in AMMSC-derived secretome and to characterize dynamic EMT states in primary AMEpiCs. To explore the potential role of AMMSCs and AMMSC-derived secretome in AM pathogenesis, we then co-transplanted AM-1 cells with primary AMMSCs or transplanted AM-1 cell pretreated with AMMSC-derived secretome into the jawbone of immunocompromised mice and observed the intraosseous AM tumor growth. Our results demonstrate that AMMSCs promote EMT and orthotopic AM tumor growth via their secretome. In addition, co-transplantation with AMMSCs facilitated the formation of CD31-positive vascular-like structures between tumor epithelial islands. This study has established a stromal cell-facilitated orthotopic AM model that mimics the intraosseous tumor microenvironment, a useful platform that enables further exploring the cellular and molecular mechanisms underlying AM pathogenesis in the jawbone and identifying potential therapeutic targets for AM treatment.

## Results

### AMMSC-derived secretome primes tumor epithelial cells to form subcutaneous xenograft

To explore the direct and indirect roles of tumor-associated stromal cells on the tumor epithelial cells, we isolated primary MSCs from AM tissues (AMMSCs) and ultrafiltered (30 kDa) the conditioned medium of AMMSCs as AMMSC-derived secretome, which was enriched with extracellular vesicles (EVs) as demonstrated by western blot analysis of the expression of several EV-specific markers (CD63, CD81, and CD9) (Figure S1A), nanoparticle tracking analysis (NTA) (Figure S1B), scanning electron microscopy (Figure S1C), flow cytometric analysis (Figures S1D and S1E), and functional uptake assay (Figure S1F and Video S1). The information about the primary AM cells utilized in this study is detailed in Table S1.

**Figure 1:**
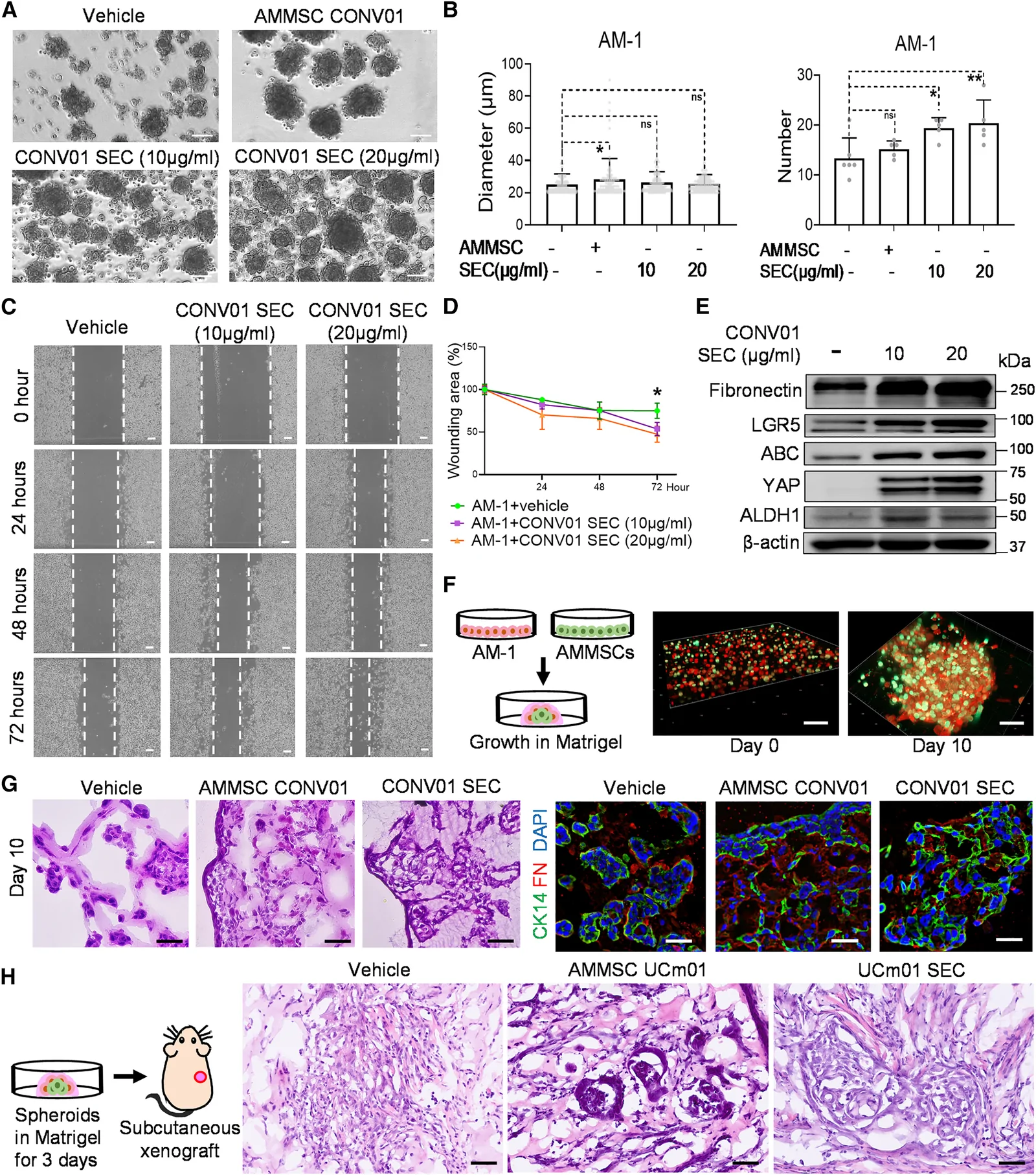
AMMSC-derived secretome enhances 3D-sphere formation, migration, and subcutaneous xenograft tumor formation by AM-1 cells (A) Sphere formation of AM-1 cells combined with AMMSC CONV01 or AMMSC CONV01-derived secretome (SEC) for 10 days. Scale bars, 20 μm. Representative images of three independent experiments. See also Figures S2A and S2B. (B) Quantification of sphere number and size in (A). Data are mean ± SD. One-way ANOVA, Dunnett’s multiple comparisons test, *n* = 3 technical replicates from 3 independent experiments. ns, not significant, ∗*p* < 0.05 and ∗∗*p* < 0.01. See also Figures S2A and S2B. (C) Wound healing assay of migrating AM-1 cells treated with AMMSC CONV01-derived SEC. Scale bars, 30 μm. Representative images of four independent experiments. See also Figure S2C. (D) Quantification of wound area in (C). Data are mean ± SD. Two-way ANOVA, Tukey’s multiple-comparisons test, *n* = 3 technical replicates from four independent experiments. ∗*p* < 0.05. See also Figure S2C. (E) Expression of EMT-related genes in AM-1 cells treated with AMMSC-derived SEC for 3 days. Blots are representative of three independent experiments using SECs from three biological donors (CONV01, CONV02, and CONV04). See also Figure S2D. (F) Representative confocal images of AM-1 cells (labeled in red) combined with AMMSCs CONV01 (labeled in green) in matrigel. Scale bars, 100 μm. Two independent experiments were conducted. (G)Upper left: H&E staining of tumor spheroids. Immunofluorescence study showed the expression of the odontogenic marker, CK14, EMT marker, and fibronectin (FN) in tumor spheroids on day 10. Scale bars, 50 μm. Representative images of two independent experiments from each strain (two biological replicates, CONV01 and UCm01). (H) The spheroids were formed with AM-1 cells alone, AM-1 cells combined with AMMSC CONV01, or AM-1 cells pretreated with AMMSC CONV01-derived SEC (20 μg/mL) for 3 days and subcutaneously transplanted to the flank of nude mice for 1 month. The H&E staining of the subcutaneous xenograft. Scale bars, 50 μm. Representative images are shown from four mice per group.

Video S1. Uptake of AMMSC CONV01-derived secretome by AM-1 cells, related to Figure 1

We then evaluated the effect of AMMSCs and AMMSC-derived secretome on formation of 3D spheroids, migration, and generation of subcutaneous xenograft tumors by AM epithelial cells. The results revealed that AM-1 cells (a human AM cell line) cocultured with AMMSCs (1:1) formed larger 3D spheroids than those formed by the same cell number of AM-1 cells (Figure 1A; Figures S2A and S2B). Meanwhile, stimulation with AMMSC-derived secretome significantly promoted AM-1 cells to form 3D spheroids as evidenced by an increased size and numbers of spheroids compared to the control (Figures 1A and 1B; Figures S2A and S2B). In addition, the EV-enriched secretome derived from AMMSCs not only promoted AM-1 migration (Figures 1C and 1D; Figure S2C and Video S2) but also upregulated the expression of several EMT- and stem cell-related genes, such as fibronectin, LGR5, active β-catenin (ABC), YAP, and ALDH1 (Figure 1E; Figure S2D).

Video S2. Wound healing assay of AM-1 treated with AMMSC CONV01-derived secretome, related to Figure 1

Next, we observed the spatial interaction between AMMSCs and AM-1 epithelial cells by 3D culture in matrigel. Our results showed that AMMSCs infiltrated into AM-1 epithelial cell clusters within the tumor spheroids (Figure 1F) and facilitated the formation of distinct architecture within the tumor epithelial islands compared with spheroids formed by AM-1 cells alone (Figure 1G). Notably, stimulation with AMMSC-derived secretome also promoted the formation of anastomosis between epithelial islands within the spheroids generated by AM-1 cells (Figure 1G). *In vivo*, we observed that subcutaneous co-transplantation of AM-1 cells with AMMSCs or AM-1 cells alone pretreated with AMMSC-derived secretome generated tumor-like xenografts compared to control AM-1 cells without pretreatment with secretome, which displayed loosely, unarranged epithelial cells in the residual structure (Figure 1H). Collectively, our results have demonstrated that both AMMSCs and their secretome could facilitate *ex vivo* tumor spheroids formation and subcutaneous xenograft AM tumor formation in nude mice, suggesting that both direct cell-to-cell interactions and indirect communication via AMMSC-derived EV-enriched secretome play critical roles in facilitating AM tumor formation.

### Unique miRNA signature in AMMSC-derived secretome upregulates EMT-related genes

**Figure 2:**
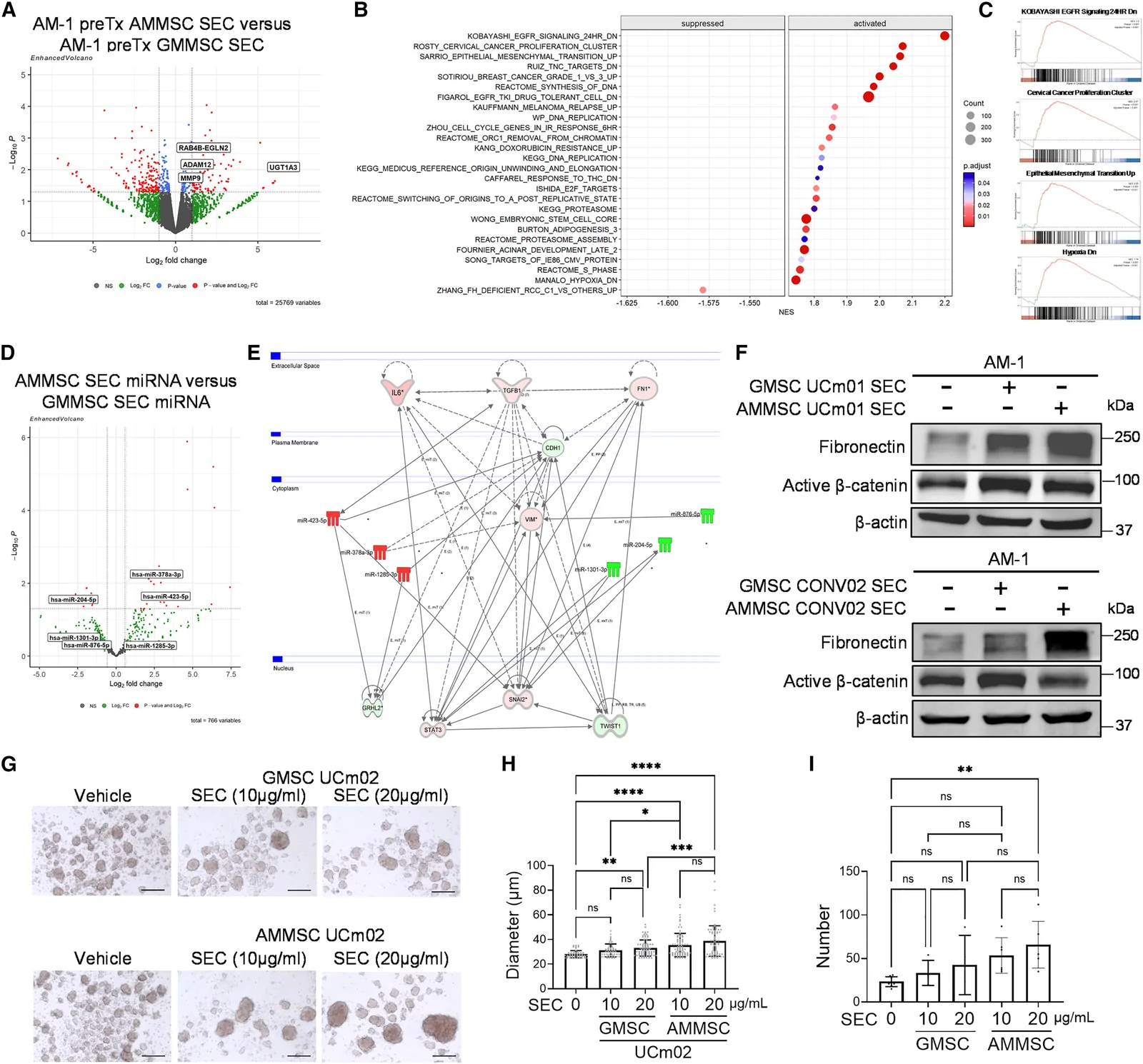
Unique microRNA signature in AMMSC-derived secretome that targets the expression of EMT-related genes (A) Volcano plot of differentially expressed genes (DEGs) from mRNA sequences of AM-1 cells treated with AMMSC-derived secretome (SEC) compared to those treated with GMSC-derived SEC from the same patient (*n* = 3 biological replicates, CONV01, CONV02, and CONV03). (B) Hallmark pathways normalized enrichment score (NES) from gene set enrichment analysis (GSEA). See also Figures S3A–S3C. (C) Representative GSEA enrichment plots. (D) Volcano plot of microRNA profile in AMMSC-derived SEC (*n* = 3 biological replicates, CONV01, CONV02, and CONV04) compared with that in GMSC-derived SEC (*n* = 2 biological replicates, GMSC01 and GMSC02). (E) Network analysis integrated mRNA and microRNA sequences via QIAGEN Ingenuity Pathway Analysis (IPA). (F) Expression of stemness marker, active β catenin (ABC), and EMT marker, fibronectin (FN), in AM-1 cells treated with AMMSC-derived SEC or GMSC-derived SEC for 3 days (*n* = 2 biological replicates, UCm01 and CONV02). See also Figure S3D. (G) Sphere formation of AM-1 cells treated with AMMSC UCm02-derived SEC or GMSC UCm02-derived SEC for 10 days. Scale bars, 100 μm. Representative images of three independent experiments. See also Figures S4A and S4B. (H) Quantification of sphere size in (G). Data are mean ± SD. One-way ANOVA, Tukey’s multiple-comparisons test. *n* = 3 technical replicates from three independent experiments. ns, not significant, ∗*p* < 0.05, ∗∗*p* < 0.01, ∗∗∗*p* < 0.001, and ∗∗∗∗*p* < 0.0001. See also Figures S4A and S4B. (I) Quantification of sphere number in (G) and Figure S4A. Data are represented as mean ± SD from two biological replicates (UCm02 and CONV03) with three to six technical replicate wells per condition for each biological replicate. One-way ANOVA; Tukey’s multiple-comparisons test. ns, not significant; ∗∗*p* < 0.01. See also Figures S4A and S4B.

To determine whether AMMSCs are distinctive from normal oral tissue-derived MSCs, we first performed bulk mRNA sequencing to compare the gene expression profiles in AM-1 cells following treatment with secretomes derived from AMMSCs and gingival-derived MSCs (GMSCs) from the same AM patients (Tables S1 and S2). The volcano plot showed upregulated genes in AM-1 cells treated with AMMSC-derived secretome compared to those treated with GMSC-derived secretome (Figure 2A). Hallmark pathways normalized enrichment score (NES) from gene set enrichment analysis (GSEA)^17^ showed upregulated pathways related to EGFR-regulating genes, proliferation, and EMT, and downregulated genes in hypoxia (Figures 2B and 2C; Figures S3A–S3C).

Next, we performed miRNA sequencing to compare the miRNA profiles in the secretome derived from AMMSCs, and GMSCs derived from healthy subjects. The volcano plot showed that the secretome of AMMSCs contained more EMT-regulated miRNAs than that from normal GMSCs (Figure 2D). We further performed QIAGEN Ingenuity Pathway Analysis (IPA) by integrating the mRNA sequence of AM-1 cells (Figures 2A–2C) and miRNA sequence of the secretome (Figure 2D). The network analysis showed that the upregulated mir-423-5p, mir-378a-3p, and 1285-3p in AMMSC-derived secretome are able to inhibit the expression of GRHL2 and CDH1 genes, while the downregulated mir-876-5p, mir-204-5p, and mir-1301-3p in AMMSC-derived secretome are able to enhance the expression of STAT3, SNAI2, VIM, and FN1 genes in AM-1 cells (Figure 2E). Western blot analysis confirmed that treatment with AMMSC-derived secretome promoted fibronectin expression and decreased E-cadherin expression in AM-1 cells (Figure 2F; Figure S3D). In addition, AMMSC-derived secretome enhanced spheroid formation of AM-1 cells compared with GMSC-derived secretome (Figures 2G–2I; Figures S4A and S4B), whereas its migration-promoting effect was comparable with that of GMSC-derived secretome (Figures S4C and S4D). These results have demonstrated a unique signature of functional miRNA network in EV-enriched secretome derived from AMMSC that promotes the EMT process in AM epithelial cells.

### Single-nucleus ATAC and RNA sequencing reveals a dynamic EMT trajectory of AM epithelial cells activated via AMMSC-derived secretome

**Figure 3:**
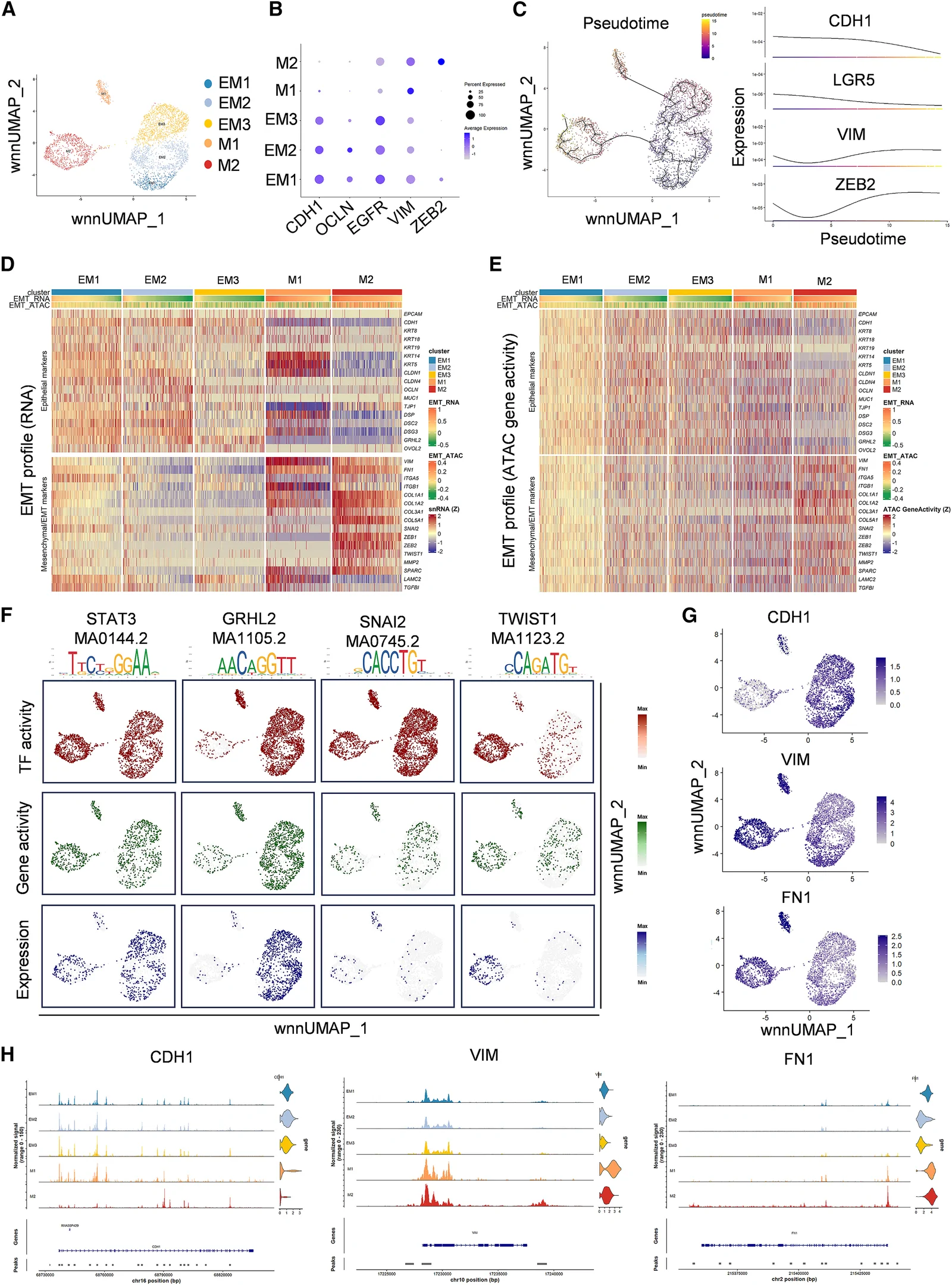
Single-nucleus multiome profiling reveals a dynamic EMT trajectory in primary ameloblastoma epithelial cells (A) The weighted-nearest neighbor (WNN) integration of 10 × multiome single-nucleus ATAC and RNA data from primary AM epithelial cells (CONV02; *n* = 3561 nuclei) treated with its AMMSC-derived secretome (20 μg/mL, 72 h). EM, epithelial to mesenchymal status; M, mesenchymal status. (B) Dot plot of the RNA expression in each cluster. (C) Pseudotime trajectory from EM1 to M1 or M2. The RNA expression of stem cell gene LGR5 and EMT-related genes CDH1, VIM, and ZEB2 in pseudotime. (D) Heatmap of expression EMT-related gene RNA level in each cluster. For each cluster, cells were downsampled to 300 and ordered within clusters (EM1–EM3, M1 and M2) by decreasing EMT_RNA module score. (E) Matched heatmap of chromatin accessibility (gene activity) of EMT-related genes in each cluster. (F) WNN uniform manifold approximation and projection (UMAP) features maps of transcription factor motif activity (chromVAR), ATAC gene activity, and RNA expression for STAT3, GRHL2, SNAI2, and TWIST1. (G) Feature plots showing RNA expression of CDH1, VIM, and FN1 across the WNN UMAP. (H) snATAC-seq genomic tracks along denoting chromatin accessibility peaks and RNA levels of CDH1, VIM, and FN1 with violins indicating gene expression across EM1–EM3 and M1 and M2.

To further characterize dynamic EMT states in primary AM epithelial cells (AMEpiCs), we performed single-nucleus ATAC and RNA sequencing of primary AMEpiCs (CONV02) treated with the AMMSC-derived secretome (from the same patient). The weighted-nearest neighbor (WNN) analysis integrated ATAC and RNA data from single nucleus of AMEpiCs (*n* = 3561) and clustered them into five groups, EM1 (EMT), EM2, EM3, M (mesenchymal)1, and M2, respectively (Figure 3A).^18^ The dot plot showed EMT-related features in each cluster, and three EM groups (EM1, EM2, and EM3) expressed higher epithelial markers, ECAD and OCLN. In contrast, two mesenchymal (M1 and M2) groups expressed extremely low levels of ECAD and OCLN (Figure 3B). Of note, both EM1 and M2 clusters expressed higher ZEB2. Pseudotime analysis displayed a dynamic EMT-related gene expression (Figure 3C). During the pseudotime trajectory, CDH1 decreased, while VIM and ZEB2 increased. Interestingly, at the beginning, EM1 showed activated VIM and ZEB2, with high CDH1 and the EMT intermediate marker LGR5.^16^ Then, EM2 showed a transition of decreased VIM and ZEB2 and elevated in EM3 to M status. Random sampling (*n* = 300) of the single nucleus sequence in each cluster showed higher expression of epithelial-related genes in EM1 and EM2 and higher expression of mesenchymal/EMT-related genes in M2 (Figure 3D). In addition, both epithelial- and mesenchymal/EMT-related genes in EM1 showed higher RNA expression and higher chromatin accessibility (Figures 3D and 3E). We used chromVAR to estimate motif activity within ATAC peaks,^19^ and EM1, EM2, and EM3 clusters showed higher GRHL2 chromatin accessibility and RNA expression, as well as higher GRHL2 motif activity. In comparison, M2 exhibited higher TWIST1 chromatin accessibility and RNA expression, as well as higher TWIST1 motif activity (Figure 3F). However, EM1, EM2, and EM3 showed higher STAT3 chromatin accessibility and RNA expression, and M2 displayed slightly higher STAT3 motif activity. Similarly, SNAI2 showed higher gene expression in EM1, EM2, and M2, but estimated SNAI2 transcription factor activity was lower in M2 compared to the three EM clusters and M1. Feature plots and ATAC peaks showed three EM clusters with higher gene expression and chromatin accessibility in CDH1, while M1 and M2 showed higher gene expression and chromatin accessibility in VIM (Figures 3G and 3H). FN1 displayed higher chromatin accessibility and gene expression in early EM1 and two M clusters. Taken together, our results suggest a dynamic EMT trajectory in primary AMEpiCs treated with AMMSC-derived secretome.

### Co-transplantation of human AMMSCs in AM spheroids facilitates orthotopic tumor formation with recapitulated histological features of human AM

**Figure 4:**
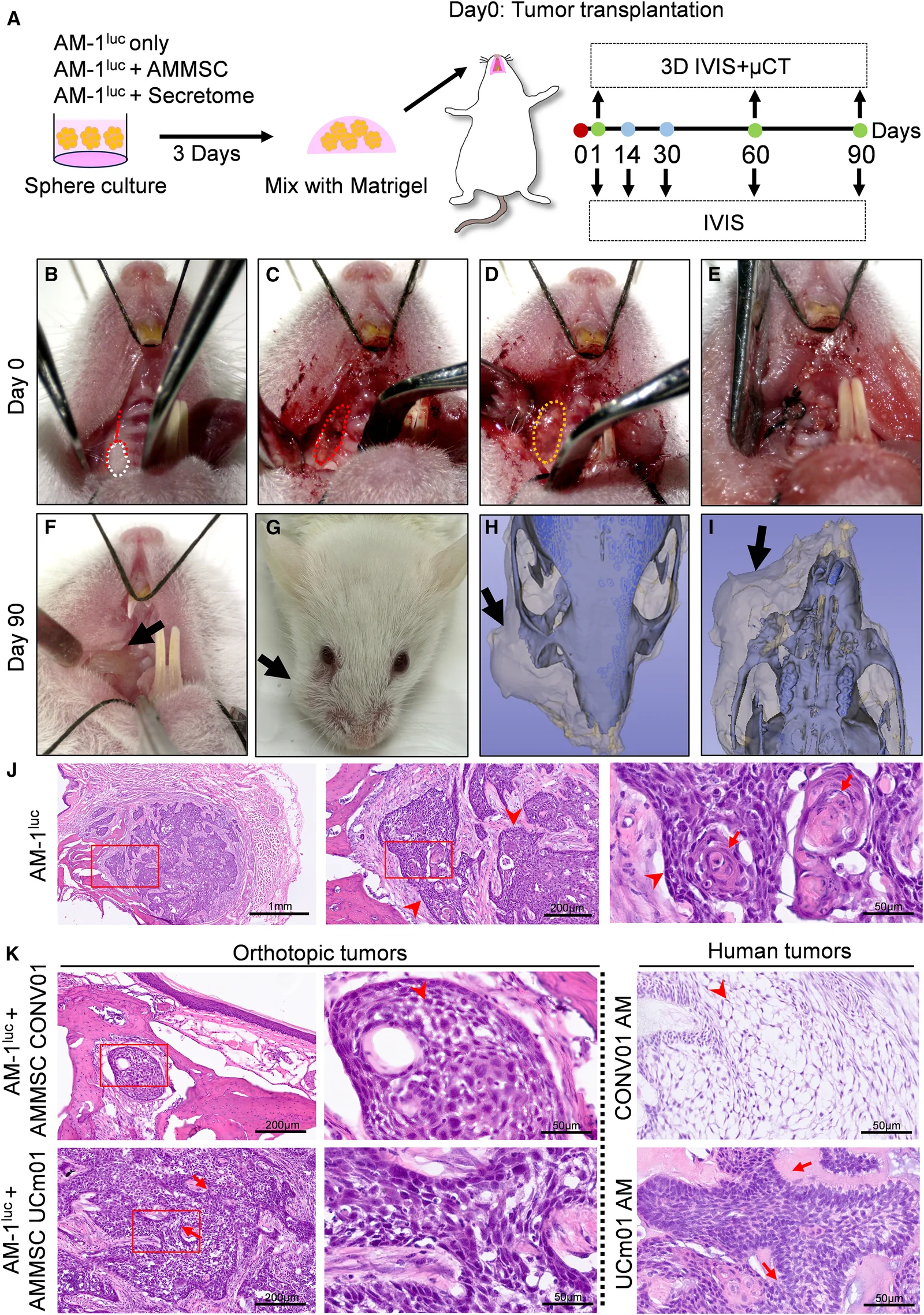
Establishment of intraosseous orthotopic mouse model of human ameloblastoma (A) Schematic diagram of cell preparation and the *in vivo* observation timeline. AM-1^luc^ cells (1.2 × 10^6^), or AM-1^luc^ and AMMSC (0.6 × 10^6^/each, 1:1 ratio), or AM-1^luc^ (1.2 × 10^6^) treated with AMMSC-derived secretome (20 μg/mL) were cultured as spheroids for 3 days and mixed with matrigel before transplantation. Tumor growth was monitored using an *in vivo* imaging system (IVIS) and micro-CT over 90 days. (B–E) Representative images of surgical procedure for tumor transplantation into the mouse maxilla. (B) The incision line (red dotted line) was made along the maxillary ridge, anterior to the first molar (white dotted line). (C) After reflecting the flap, a bony cavity was prepared using surgical burs (red dotted circle). (D) The mixture of tumor spheroids and matrigel was transplanted into the bony cavity (yellow dotted circle). (E) The wound was primarily closed with 7-0 silk sutures. (F) Representative intraoral photograph taken 3 months post-surgery, showing complete wound healing with mild maxillary swelling. (G) Representative external profile of the mouse 3 months after transplantation with visible swelling at the tumor site. (H and I) Representative reconstructed micro-CT images display bone (blue) and soft tissue (gray). Erosion of the maxillary bone, including the zygoma, along with soft tissue swelling was observed. (J) Left: low magnification of H&E staining. Scale bars, 1 mm. Middle: higher magnification of the left image. Scale bars, 200 μm. Right: higher magnification of the middle image. Scale bars, 50 μm. Representative images of AM-1^luc^ cells transplanted alone formed plexiform-like ameloblastoma with palisading arrangement of the outer layer in epithelial islands (arrowhead). Keratin pearls (arrow) in the central region as acanthomatous ameloblastoma was noted. *n* = 4 mice. (K) Upper images: tumors formed by AM-1^luc^ cells co-transplanted with AMMSC CONV01 (follicular and plexiform, *n* = 2 mice) exhibited a follicular-like pattern with central loosely arranged cells resembling the stellate reticulum (arrow). Lower images: tumors generated by AM-1^luc^ with AMMSC UCm01 (plexiform type, *n* = 4 mice) retained a plexiform-like structure with anastomosing strands (arrowhead). Left: orthotopic tumors. Lower magnification of middle images. Scale bars, 200 μm. Middle: orthotopic tumors. Higher magnification of left images. Scale bars, 50 μm. Right: human tumors. Scale bars, 50 μm.

We then performed intraosseous implantation of spheroids of AM-1 cells transduced with lentiviral luciferase (AM-1^luc^), either alone, or pretreated with AMMSC-derived secretome, or in combination with AMMSCs, into the maxilla of advanced severe immunodeficiency (ASID) mice (Figures 4A–4E). After 3 months, a remarkable lateral expansion in the transplanted site of the maxilla was observed (Figures 4F–4I). On day 90, clinical gross view of the mouse maxilla with tumor cell transplantation revealed bony expansion, and the sham control group exhibited a concave profile and a flat maxillary ridge (Figure S5). Micro-CT scanning and histological analysis showed that the sham control group exhibited scarce soft tissue in the bony defect, while groups transplanted with tumor cells exhibited tumor growth within the maxilla (Figure S6). Immunofluorescence study showed abundant immunostaining signals of human mitochondria, which further confirmed the long-term survival and tumorigenic capability of transplanted human cells in the intraosseous microenvironment (Figure S7). A mildly reversed nuclear polarity, a characteristic feature of ameloblasts, was also observed, as evidenced by the localization of human mitochondria adjacent to the basement membrane with nuclei positioned oppositely (Figure S7).

**Figure 5:**
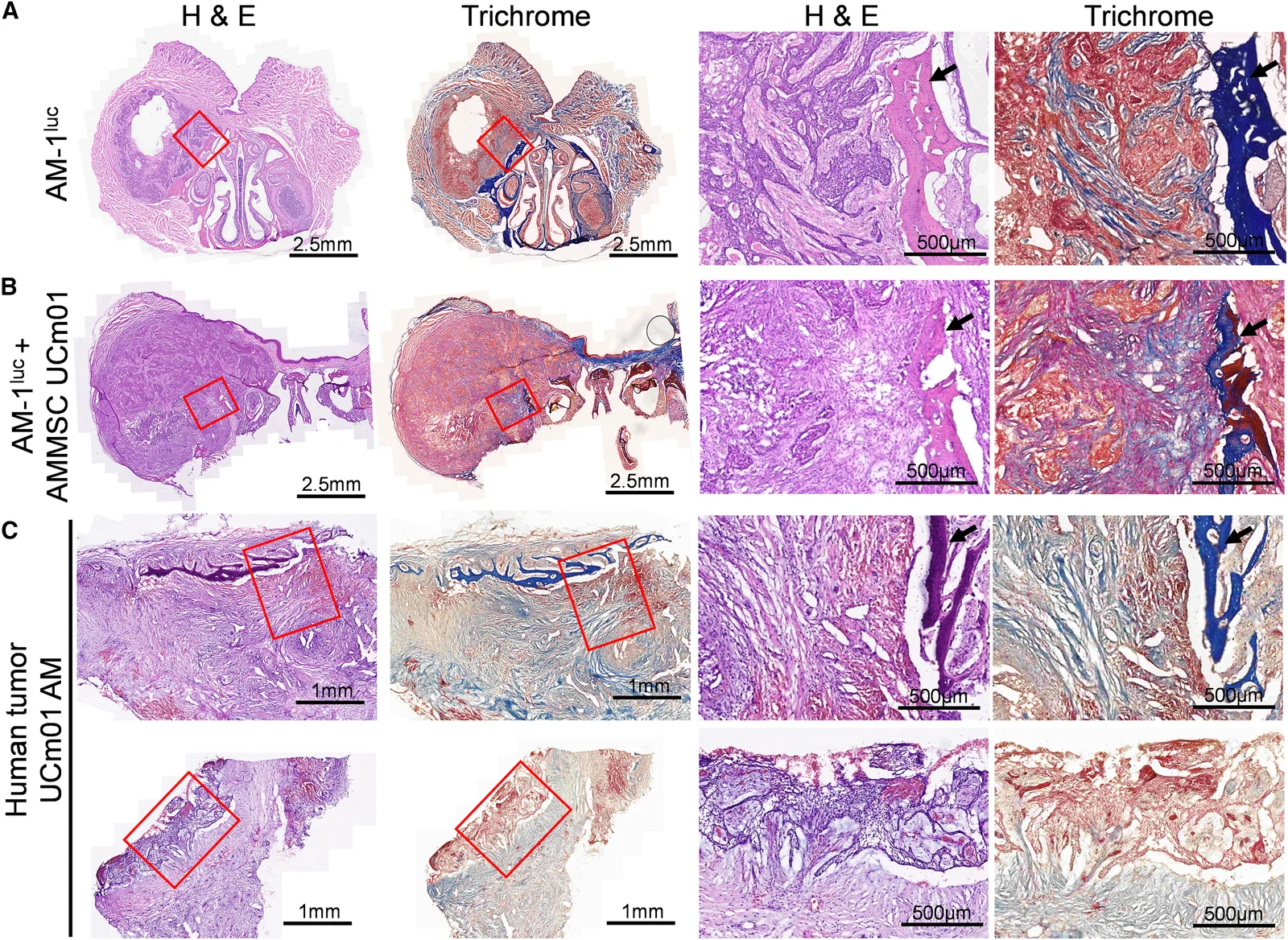
AMMSCs facilitate the reconstruction of ameloblastoma microenvironment within the mouse orthotopic model (A) AM-1^luc^ only group displayed epithelial island (red) and collagen fibers (blue) surrounding the bone structure (arrow) in the trichrome staining. Left two images: lower magnification of right two images. Scale bars, 2.5 mm. Right two images: scale bars, 500 μm. (B) AM-1^luc^ combined with AMMSC UCm01 group exhibited a diffuse connective tissue surrounding the bone structure (arrow) in the trichrome staining. Left two images: lower magnification of right two images. Scale bars, 2.5 mm. Right two images: scale bars, 500 μm. (C) UCm01 tissue showed diffuse connective tissue surrounding the bone structure (arrow) in the trichrome staining. Left four images: lower magnification of right four images. Scale bars, 1 mm. Right four images: scale bars, 500 μm. All representative images are shown from two mice per group. Two independently stained sections per tumor were examined. The human comparator represents one biological specimen from patient UCm01. See also Figure S8.

**Figure 6:**
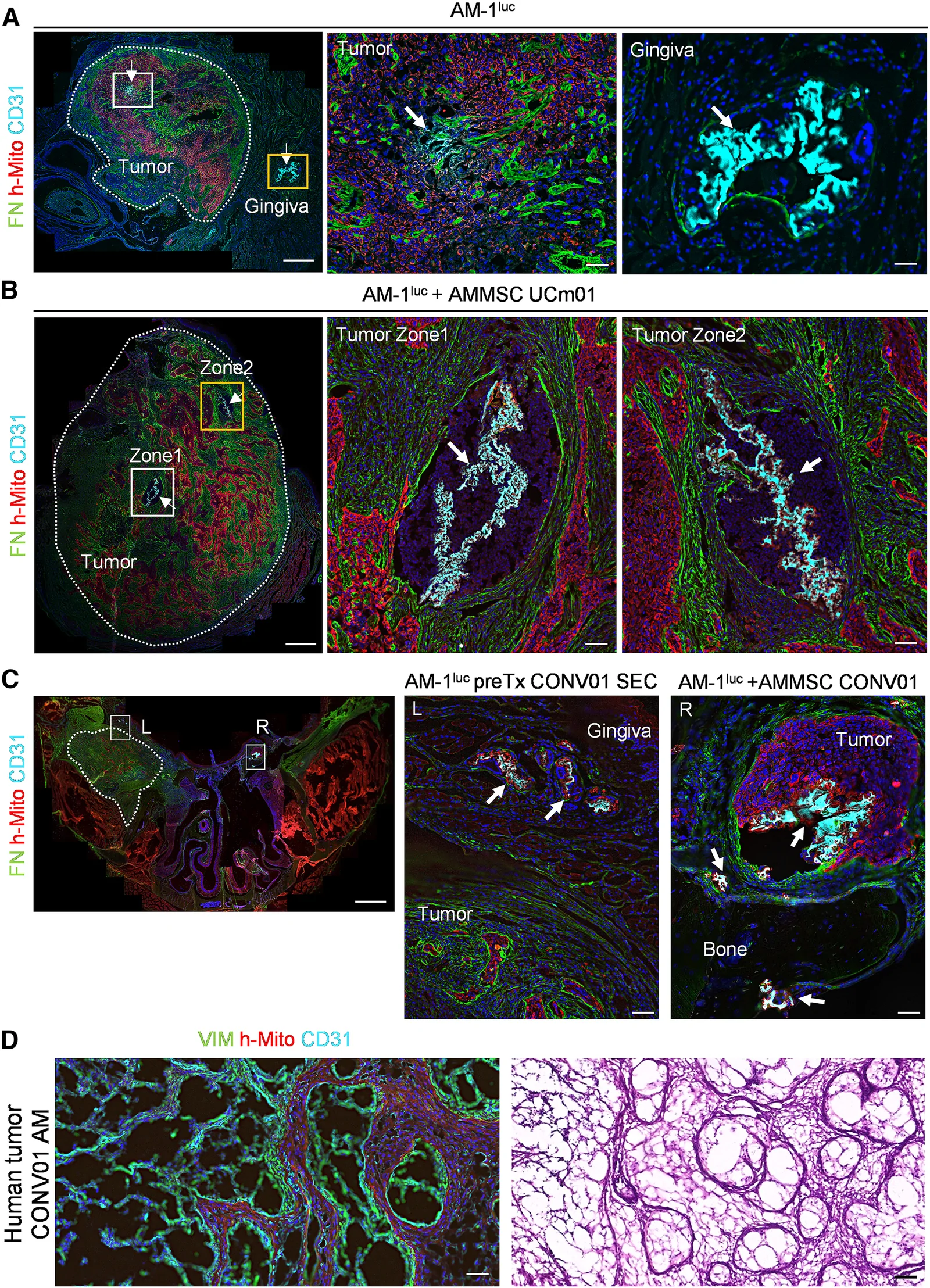
AMMSCs promote angiogenesis within the orthotopic tumors and recapitulate the pathological features of human ameloblastoma (A) AM-1^luc^ only group showed CD31^+^h-Moto^−^ vascular-like structure in the subgingival connective tissue area and limited CD31 positive signal within the tumor. Left, scale bars, 500 μm. Middle and right, scale bars, 50 μm. h-Mito: human mitochondria. Representative images are shown from four mice per experimental group. (B) AM-1^luc^ combined AMMSC UCm01 group displayed large CD31^+^h-Mito^+^ vascular-like structure within the tumor microenvironment. Left, scale bars, 500 μm. Middle and right, scale bars, 50 μm. Representative images are shown from four mice per experimental group. (C) Left: cross section of the mouse that implanted spheroids of AM-1^luc^ combined AMMSC CONV01 at right maxilla, and spheroids of AM-1^luc^ pretreated with AMMSC CONV01-derived secretome for 3 days at left maxilla. Scale bars, 1000 μm. Right: AM-1^luc^ combined AMMSC CONV01 group displayed CD31^+^h-Mito^+^ vascular-like structure within the tumor and close to the bone. Middle: AM-1^luc^ pretreated with AMMSC CONV01-derived secretome group showed CD31^+^h-Moto^+^ vascular-like structure in the subgingival connective tissue area, but no visible CD31 signal within the tumor. Middle and right images: scale bars, 50 μm. Representative images are shown from two mice per experimental group. (D) Left: human ameloblastoma tissues showed abundant CD31^+^h-Mito^+^ vascular-like structure within the tumor. Right: H&E staining. Scale bars, 50 μm. Representative images of the human sample of CONV01.

To further evaluate the histological characteristics of the orthotopic AM tumors, we compared their tissue architecture with that of human AM. Tumor epithelial islands formed by AM-1^luc^ cells alone were outlined by cuboidal cells and contained central foci of keratin formation, consistent with features of acanthomatous AM. (Figure 4J). Although it did not distinctly resemble the plexiform subtype of conventional AM, the orthotopic tumor formed by AM-1^luc^ cells still displayed histological features comparable to the original plexiform of AM tissue from which the AM-1 cell line was derived.^20^ Tumor islands formed by spheroids of AM-1^luc^ cells co-transplanted with AMMSC exhibited conventional AM features including the follicular-like pattern, with slightly loosely arranged cells resembling the stellate reticulum and anastomosing architectures (Figure 4K). Furthermore, Trichrome staining revealed a diffuse stromal collagen deposition and a few bundles peripherally to the orthotopic tumors and the surrounding bone in the AM-1^luc^ combined with AMMSC UCm01, resembling the original AM tissue (Figure 5; Figure S8). Interestingly, regardless of orthotopic tumor size, only tumors formed by combined transplantation with AMMSCs harbored CD31^+^h-mitochondria^+^ vascular-like structures within the tumor epithelial islands (Figures 6B and 6C). By contrast, the AM-1^luc^ alone group showed CD31^+^ vascular-like structures mainly outside the tumor, with limited intratumoral CD31^+^ signals (Figure 6A). Orthotopic tumors generated from AM-1^luc^ cells pretreated with AMMSC-derived secretome also exhibited limited intratumoral CD31^+^ signals (Figure 6C). In the human AM sample, CD31^+^h-mitochondria^+^ signals were detected within vascular-like structures in the tumor epithelial islands (Figure 6D). Collectively, these findings suggest that AMMSCs play a critical role in orchestrating intraosseous AM tumor growth with recapitulated histopathological features characteristic of their parental AM tissues.

### AM epithelial cells primed by AMMMSC-derived secretome acquire enhanced tumorigenic potentials in the intraosseous microenvironment

**Figure 7:**
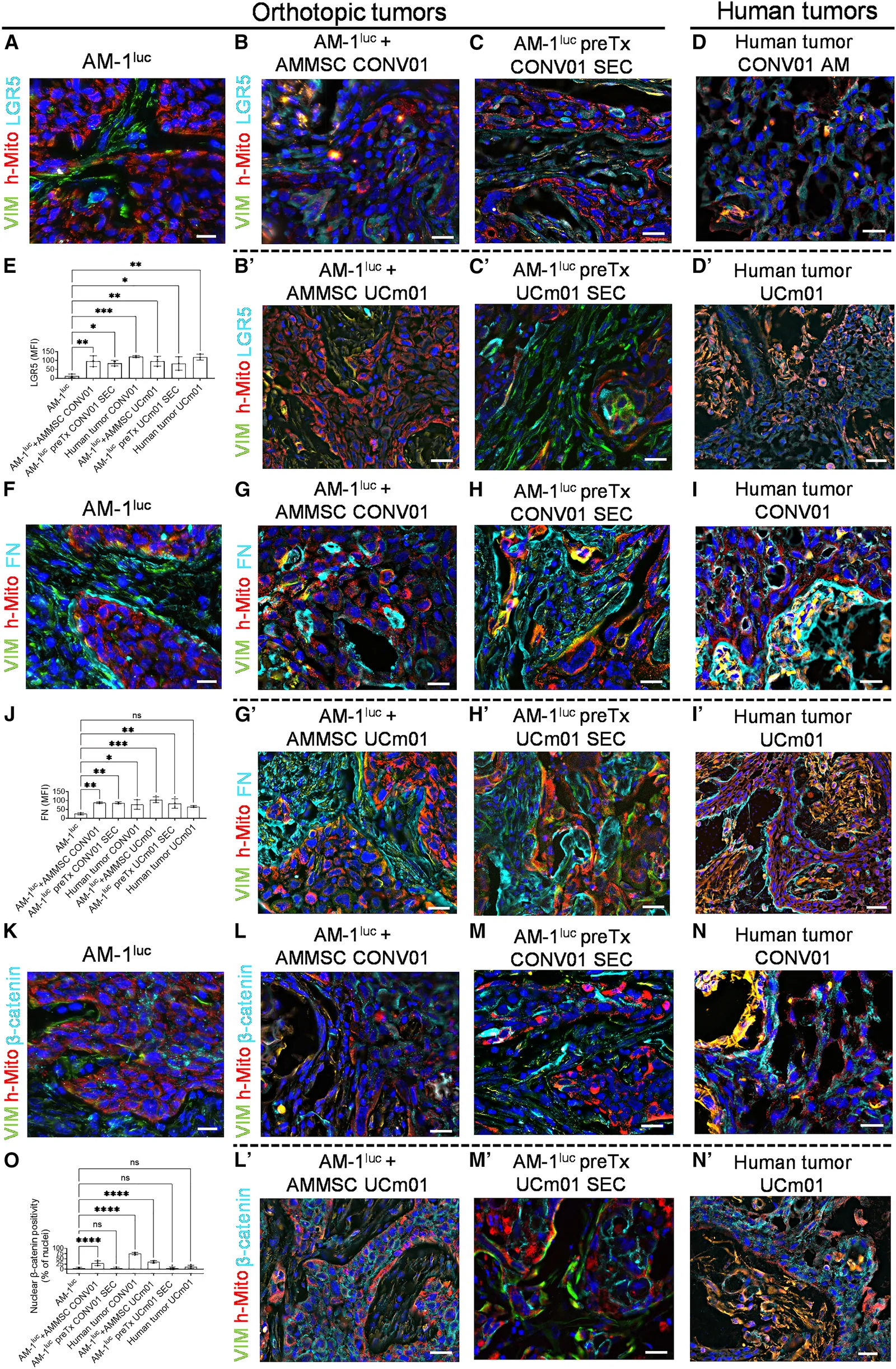
Orthotopic tumors recapitulate the histological and molecular features of ameloblastoma (A), (F), and (K) The AM-1^luc^-only group exhibited limited colocalization of human mitochondria (red) and vimentin (green) at the borders of epithelial islands. (B), (G), and (L) The AM-1^luc^ combined with primary AMMSC CONV01 group. (B′), (G′), and (L′) The AM-1^luc^ combined with primary AMMSC UCm01 group. (C), (H), and (M) The AM-1^luc^ pretreated with AMMSC CONV01-derived secretome group. (C′), (H′), and (M′) The AM-1^luc^ pretreated with AMMSC UCm01-derived secretome group. (D), (I), and (N) The immunofluorescence study of the original human tumor of CONV01. (D′), (I′), and (N′) The immunofluorescence study of the original human tumor of UCm01. Scale bars: 20 μm. (E), (J), and (O) Quantification of the mean of fluorescence intensity (MFI) of LGR5, fibronectin (FN), and nuclear β-catenin positivity in orthotopic tumors and human tumors. Data are represented as mean ± SD from two mice per group. Five randomly selected fields were analyzed per section. One-way ANOVA followed by Tukey’s multiple-comparisons test. ns, not significant, ∗*p* < 0.05, ∗∗*p* < 0.01, ∗∗∗*p* < 0.001, and ∗∗∗∗*p* < 0.0001.VIM, vimentin; h-Mito, human mitochondria; FN, fibronectin.

We then performed immunofluorescence staining of tumor tissue sections from intraosseous tumors formed by transplantation of either AM-1^luc^ cells alone (Figures 7A, 7F, and 7K), or AM-1^luc^ cells combined with AMMSCs (Figures 7B, 7G, and 7L) and AM-1^luc^ cells primed by AMMSC-derived secretome (Figures 7C, 7H, and 7M). The results showed that all intraosseous tumor tissues harbored positive immunoreactive signals of vimentin, LGR5, ABC, and fibronectin, which are hallmark molecular markers of human AM.^16^ Overall, LGR5 expression were more prominent in the center of the epithelial islands (Figures 7A–7D), while fibronectin-positive cells located in the stromal region between the tumor islands, with the strongest signal closely adjacent to the tumor epithelium (Figures 7F–7I). The expression of β-catenin was observed both surrounding and within the nuclei of the tumor epithelium (Figures 7K–7N). AM-1^luc^ cells were identified by positive immunostaining of human mitochondrial markers (red), while human primary AMMSCs were distinguished by the colocalization of human mitochondria (red) and vimentin (an MSC marker) expression (green), appearing as an overlapped yellow color (Figure 7). In tumors formed by AM-1^luc^ cells combined with AMMSCs, vimentin-positive human cells (yellow) were observed in the stromal regions and in part of the outer layer of epithelial islands (Figures 7B, 7G, and 7L). Interestingly, in tumors formed by AM-1^luc^ cells alone (Figures 7A, 7F, and 7K) or AM-1^luc^ cells pretreated with AMMSC-derived secretome (Figures 7C, 7H, and 7M), vimentin-positive human cells (yellow) were also observed at the border of the epithelial islands, suggesting that transplanted AM-1^luc^ cells may have undergone EMT during the formation of orthotopic tumors. Moreover, tumors formed by AM-1^luc^ combined with AMMSCs or pretreated with AMMSC-derived secretome exhibited higher expression levels of LGR5, nuclear non-phospho β-catenin, and fibronectin, resembling that of human specimens, compared with tumors derived from AM-1^luc^ cells alone (Figures 7E, 7J and 7O).

**Figure 8:**
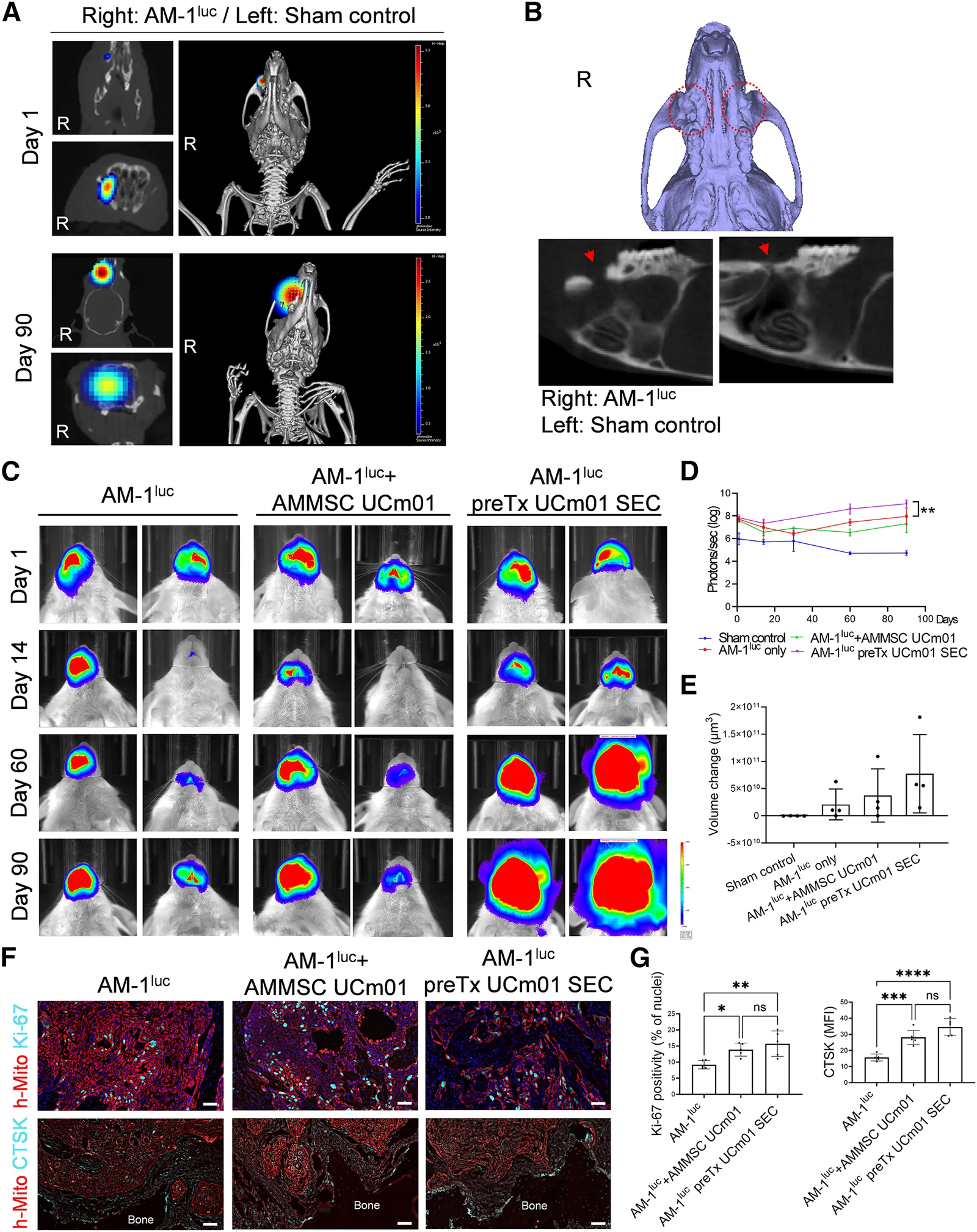
Longitudinal IVIS and micro-CT monitoring of orthotopic tumors (A) An ASID mouse was transplanted with AM-1^luc^ cells at the right maxilla and matrigel at the left maxilla (sham control). Co-registration of 3D IVIS and micro-CT on days 1 and 90. (B) Representative reconstructed and sagittal view images of micro-CT from the mouse in (A) on day 90. A radiolucent lesion was observed at the tumor site, whereas the sham control showed a healed bony structure. See also Figures S6 and S8. (C) Bioluminescence signals of three groups: AM-1^luc^ only, AM-1^luc^ combined with AMMSC UCm01, and AM-1^luc^ pretreated with AMMSC UCm-derived secretome (*n* = 4 mice per group). (D) The luminescence intensity of photons emitted from each tumor was quantified. Data are mean ± SD. Two-way ANOVA with mixed-effects modeling, followed by Tukey’s multiple-comparisons test. *n* = 4 mice per group. *p* < 0.0001 for comparisons between AM-1^luc^ only vs. sham control, AM-1^luc^ combined with AMMSC UCm01 vs. sham control and AM-1^luc^ pretreated with AMMSC UCm01-derived secretome vs. sham control. No significant difference was observed between AM-1^luc^ and AM-1^luc^ combined with AMMSC UCm10. ∗∗*p* < 0.01 for the comparison between AM-1^luc^ pretreated with AMMSC UCm01-derived secretome vs. AM-1^luc^ only. (E) Change of tumor volume in micro-CT on day 90 compared with day 1. *n* = 4 mice per group. (F) The immunofluorescence study. VIM, vimentin; h-Mito, human mitochondria. CTSK: cathepsin K. Scale bars, 50 μm. Representative images of two independent experiments. (G) Quantification of the positive Ki-67 percentage and mean of fluorescence intensity (MFI) of CTSK in (F). Data are represented as mean ± SD from four mice per group. Five randomly selected fields were analyzed per section. One-way ANOVA followed by Tukey’s multiple-comparisons test. ns, not significant, ∗*p* < 0.05, ∗∗*p* < 0.01, ∗∗∗*p* < 0.001, and ∗∗∗∗*p* < 0.0001.

To assess the transplantation outcome and long-term growth of AM-1^luc^ cells in the mouse maxilla, we conducted 3D *in vivo* imaging system (IVIS) combined with micro-CT 1 day post-surgery, with follow-up imaging on days 14, 30, 60, and 90. The tumor spheroid transplantation site exhibited strong bioluminescence co-registered with the micro-CT images, whereas the sham control group showed no signal (Figure 8A). Micro-CT images further identified some fenestrations in the regions of aggressive bony expansion, resembling the clinical feature of AM (Figures 8B, S6, and S9). On day 90, the luminescence intensity in three experimental groups is significantly greater than that in the sham control group (*p* < 0.0001) (Figures 8C and 8D). Even though the initial cell number of AM-1^luc^ cells in spheroids formed by combination of AM-1^luc^ cells and AMMSCs was half of that in spheroids formed by AM-1^luc^ cells alone, the luminescence intensity observed in the AM-1^luc^ alone group was slightly higher but with no statistical significance compared to that of the combination group, suggesting that AMMSCs probably promoted the proliferation of co-transplanted AM-1^luc^ cells *in vivo*. Additionally, AM-1^luc^ cells pretreated with AMMSC-derived secretome showed enhanced tumorigenic potentials as evidenced by stronger bioluminescence intensity compared with AM-1^luc^ only group (*p* < 0.01, Figure 8D). However, micro-CT analysis revealed no significant differences in tumor volume changes among the three tumor groups (Figure 8E). We further evaluated bone invasion and tumor cell proliferation using cathepsin K and Ki-67 staining, respectively. Compared with the AM-1^luc^ alone group, AM-1^luc^ cells co-transplanted with AMMSCs or pretreated with AMMSC-derived secretome exhibited an increased percentage of Ki-67-positive cells within tumor islands, together with enhanced cathepsin K signals along the adjacent bone margin (Figures 8F and 8G). These results suggest that AMMSC-derived secretome plays a crucial role in AMMSC-driven orthotopic AM tumor growth in the intraosseous microenvironment.

## Discussion

In the present study, we reported the successful establishment of an intraosseous orthotopic mouse model of human AM by transplantation of 3D spheroids formed by luciferase-labeled AM-1^luc^ cells alone or in combination with primary human AMMSCs, or AM-1^luc^ cells pretreated with AMMSC-derived secretome into the maxilla of ASID mice. Findings from IVIS and live micro-CT imaging, histological analysis, and immunostaining of several key molecular markers of AM, such as vimentin, fibronectin, LGR5, and ABC, demonstrate that these intraosseous AM tumors recapitulate the major clinical and histopathological features of human AM.

Till date, the cellular and molecular mechanisms underlying the pathogenesis of AM remain largely unknown.^21^ During tooth development, ectomesenchyme receives signals from the dental lamina in the early first branchial arch epithelium and then governs the continuous initiation and organization of tooth germ formation.^22^^,^^23^ AM originates from remnants of odontogenic epithelium that form the enamel organ during tooth development, and the residual dental lamina undergoes degeneration and absorption,^24^ implying that the MSCs may play a critical role in the pathogenesis of AM. However, there is still lack of direct evidence to support this hypothesis. In the present study, the intraosseous tumors formed by transplantation of spheroids formed by mixed AM-1^luc^ cells and AMMSCs (1:1) harbored a similar luminescence intensity compared to those formed by transplantation of spheroids of AM-1^luc^ cells alone even though the later has doubled numbers of AM-1^luc^ cells compared to the co-transplantation group. Consistently, the co-transplanted AMMSC group showed a significantly higher Ki-67 index than the AM-1^luc^ alone group. This finding suggests that co-transplanted AMMSCs promote the proliferation of AM-1^luc^ cells in the intraosseous microenvironment. In addition, both the AM-1^luc^ with AMMSC co-transplantation group and the AM-1^luc^ cells pretreated with AMMSC-derived secretome showed increased cathepsin K signals along the adjacent bone margin, suggesting that AMMSCs may promote bone erosion, at least in part, through their secretome.^25^ Furthermore, we observed that only tumors formed by AM-1^luc^ cells co-transplanted with AMMSCs harbored CD31^+^human-mitochondria^+^ vascular-like structures, suggesting that a certain proportion of AMMSCs has transdifferentiated into endothelial-like cells expressing CD31.^26^ Of note, a few of CD31^+^human-mitochondria^+^ vascular-like structures were observed within the gingiva in the tumor site transplanted with AM-1^luc^ pretreated with AMMSC-derived secretome, which might be attributed to the migration of AMMMSCs from the opposite site (co-transplantation with AMMSCs) and homing to the tumor area.^15^

Through miRNA sequencing, we have identified several miRNAs in AMMSC-derived secretome that could promote the EMT process in AM epithelial cells through activating IL6/STAT3-, TGFβ-, or EGFR-mediated signaling pathways. Our results showed that both AMMSC- and GMSC-derived secretomes enhanced the expression of fibronectin and ABC of AM-1 cells. However, compared with GMSC-derived secretome, AMMSC-derived secretome exerted a stronger effect on spheroid formation, while showing a comparable effect on AM-1 cell migration. Because gingival tissue has a high regenerative and wound-healing capacity, GMSC-derived secretome may also contain pro-migratory factors that support wound closure in the AM-1 wound healing assay. Single-nucleus ATAC and RNA sequencing reveals a dynamic EMT trajectory, similar to a previous study showing an EGFR- or TGFβ-activated continuum of EMT.^27^ The pseudotime trajectory suggests the dynamic EMT process of primary AM epithelial cells is activated from a high expression of SNAI1, ZEB1/2, and TWIST1/2 in EM1. It transitions to a stable state in EM2 and EM3, decreasing the expression of these transcription factors, then elevates the expression of key EMT transcription factors, and finally loses CDH1 expression in M2. Notably, the chromatin accessibility of epithelial and EMT-related genes in EM1 status is also higher than in other statuses, suggesting that M1 may represent a poised state that expresses higher levels of LGR5. Our previous studies have shown that stromal-derived IL6 promotes AM epithelial cells to undergo EMT and may leave them in the LGR5^+^ intermediate EMT state, thereby contributing to drug resistance.^9^^,^^16^ In the present study, we found that the AMMSC-derived secretome not only promotes stemness- and EMT-related features of AM epithelial cells but also primes them to form subcutaneous and intraosseous tumors. Our results also showed that orthotopic tumors formed by AM-1^luc^ cells primed with AMMSC-derived secretome harbored colocalized immunostaining signals representative of human mitochondria and vimentin, suggesting that AM-1^luc^ cells may undergo EMT during orthotopic tumor formation. Taken together, our findings support the notion that AMMSC-derived secretome plays a critical role in AMMSC-orchestrated intraosseous AM tumor growth by promoting AM epithelial cell proliferation and EMT process. Therefore, targeting interactions between tumor epithelial and stromal cells could offer new therapeutic insights for treating this locally aggressive benign tumor.

Methodologically, AM-1^luc^ cells and AMMSCs were pre-cultured to form 3D spheroids, a common approach shown to enrich tumor stem-like cells, thereby enhancing their survival and engraftment abilities, and consequently, the tumor-forming capacity.^28^^,^^29^ One study has demonstrated the polarization of iPSC-derived early ameloblast cells in sphere culture.^30^ However, whether this approach promotes tumor formation or disrupts tumor epithelial polarization remains unclear and requires further investigation. In addition, lentiviral transduction was utilized to establish the AM-1^luc^ cell line, which might lead to the loss of certain molecular signatures for some subpopulations of tumor cells during the *in vitro* selection. To preserve tumor heterogeneity, we could transplant primarily cultured AM epithelial cells, combined with AMMSCs or tumor cells directly dissociated from tumor tissues without *ex vivo* culture. Another alternative strategy could be the use of luciferase dyes for bioluminescence imaging of transplanted primarily cultured AM cells rather than the genetic approach to generate luciferase-expressing tumor cells for transplantation.

One key challenge in creating an intraosseous model of AM lies in that it is a benign tumor that may not easily evade the surveillance of the mouse’s immune system. To reduce the immune response, we utilized ASID mice, which lack natural killer (NK) cells, NK T cells, T cells, and B cells. However, we still observed lots of immune cell infiltration at the tumor site at 2 weeks, indicating an acute innate immune response against transplanted human AM cells. Humanized mouse models may be an alternative for more accurately representing human tumor-immune interactions, despite their higher costs and extended experimental timelines. In addition, we observed a transient decrease in bioluminescence intensity within the first month post-implantation of tumor spheroids, followed by continuous tumor growth. This phenomenon might be partially explained by the adaptive survival and lagged activation of proliferation of transplanted benign AM epithelial cells in the unique bony microenvironment. Since tumor stem cells primarily exist in a quiescent state with slow cycling but can become highly proliferative upon activation by certain niche signals, thus contributing to the initiation, formation, and recurrence of tumor.^31^^,^^32^ This might provide another possible explanation for the lagged activation of proliferation of transplanted AM epithelial cells with the first month post-transplantation. Meanwhile, the early increased infiltration of residual host immune cells in ASID mice might have targeted inhibition of the transplanted human AM cells but with subpopulations of tumor stem-like cells escaping from the immune surveillance, thus contributing to the dormancy of transplanted human AM cells within the first month following transplantation.

In summary, this study has harnessed AMMSCs and their derivative secretomes to establish an intraosseous orthotopic mouse model of AM that faithfully mimics the unique intraosseous microenvironment and recapitulates the histopathological and molecular features of human AM, thus providing a clinically relevant platform to investigate AM pathogenesis and identify potential therapeutic targets.

### Limitations of the study

Given that the majority of AM originates within the jawbone, particularly, in the mandible, and develops from the first branchial arch,^33^ and considering the ease of surgical access and the larger bony volume of the mouse maxilla, we preferably selected this site for transplantation of tumor spheroids to generate the intraosseous AM model that can simulate the unique bony microenvironment of AM more accurately than the currently available subcutaneous animal models of AM. However, we could not determine whether the maxilla or mandible may contribute to distinct microenvironments for tumor pathogenesis.

In this study, we used IPA to construct a network of miRNAs and targeted mRNAs that lack direct functional interactions but still provide valuable information for further analysis. Besides, single-nucleus ATAC and RNA sequencing is based on cultured primary AM epithelial cells treated with AMMSC-derived secretome, which does not reflect the natural tumor microenvironment. In the future, more studies collecting human tumor tissues and directly dissociating them to perform single-nucleus ATAC and RNA sequencing, or single-cell RNA sequencing, could provide further insights into the tumor microenvironment. However, based on the data we provided, we could more accurately evaluate the effects of secretory factors from tumor-derived MSCs. The further study will focus on EVs or soluble proteins for targeting therapy.

## Resource availability

### Lead contact

Further information and requests for resources and reagents should be directed to and will be fulfilled by the lead contact, Ting-Han Chang (cth0616@nycu.edu.tw).

### Materials availability

This study did not generate new unique reagents.

### Data and code availability

• All raw sequencing data have been deposited to GEO (GEO: GSE317372, GSE317373, and GSE317374).
• The custom R scripts and processed snATAC+RNA data have been deposited on Zenodo (https://doi.org/10.5281/zenodo.18373021).
• Any additional information required to reanalyze the data reported in this paper is available from the lead contact upon request.

## Acknowledgments

We gratefully thank Professor Hidemitsu Harada (Iwate Medical University) for generously sharing the AM-1 cell line. We further acknowledge the Instrument Center at the National Defense Medical University for performing NanoSight analysis, scanning electron microscopy, and imaging flow cytometry, and providing access to the Tomocube system, THUNDER Imager, CLC Genomics Workbench, IVIS, and micro-CT systems. Research reported in this publication was supported by the Taiwan National Science and Technology Council (110-2314-B-A49A-553-MY3 to T.-H.C., 111-2314-B-016-018 to D.-Y.Y., 111-2629-B-016-001 to D.-Y.Y., 113-2314-B-A49-021 to T.-H.C., and 114-2314-B-A49-035 to T.-H.C.), Ministry of National Defense (MND-MAB-110-095, MND-MAB-110-123, and MND-MAB-C08-112032) (D.-Y.Y.), and Tri-Service General Hospital Research Grant (TSGH-D-110060, TSGH-E-111198, TSGH-E-112202, and TSGH-A-113003) (D.-Y.Y.).

## Author contributions

D.-Y.Y., conceptualization, methodology, investigation, funding acquisition, visualization, and writing – review and editing; C.-J.Y., C.-Y.C., and H.-N.C, investigation; C.-Y.W., methodology, validation, and writing – review and editing; J.-L.C., investigation and writing – review and editing; L.-Y.K, validation and writing – review and editing; Y.-T.C., W.-C.C., and Y.-W.C., resources; Q.Z. and A.D.L., conceptualization, supervision, and writing – review and editing; T.-H.C., conceptualization, resources, methodology, investigation, funding acquisition, formal analysis, supervision, validation, visualization, writing – original draft, and writing – review and editing. All the authors read and approved of the final manuscript.

## Declaration of interests

The authors declare no competing interests.

## STAR★Methods

### Key resources table

**Table:** 

REAGENT or RESOURCE	SOURCE	IDENTIFIER
**Antibodies**		

LGR5 Monoclonal Antibody (OTI2A2)	Invitrogen	Cat#MA5-25644; RRID: AB_2723318
LGR5 Rabbit Polyclonal Antibody	OriGene	Cat#TA324730
LGR5 Polyclonal Antibody	Invitrogen	Cat#PA5-35304; RRID: AB_2552614
APC-conjugated anti-LGR5 antibody	R&D Systems	Cat#FAB8078A; RRID: AB_3652856
Non-phospho (Active) β-catenin	Cell Signaling Technology	Cat#8814; RRID: AB_11127203
Fibronectin	Sigma	Cat#F3648; RRID: AB_476976
YAP (D8H1X) Rabbit Monoclonal Antibody	Cell Signaling Technology	Cat#14074; RRID: AB_2650491
Purified Mouse Anti-ALDH, Clone 44/ALDH (RUO)	BD biosciences	Cat#611194; RRID: AB_2224312
E-Cadherin (24E10) Rabbit Monoclonal Antibody	Cell Signaling Technology	Cat#3195; RRID: AB_2291471
CD63 Monoclonal Antibody (Ts63)	Invitrogen	Cat#10628D; RRID: AB_2532983
CD9 Monoclonal Antibody (Ts9)	Invitrogen	Cat#10626D; RRID: AB_2532982
CD81 Monoclonal Antibody (M38)	Invitrogen	Cat#10630D; RRID: AB_2532984
Cytokeratin 14 Monoclonal Antibody (LL002)	Invitrogen	Cat#MA5-11599; RRID: AB_10982092
β-actin	Santa Cruz	Cat#sc-47778; RRID: AB_626632
Goat anti-rabbit IgG-HRP	Santa Cruz	Cat#sc-2004; RRID: AB_631746
Goat anti-mouse IgG-HRP	Santa Cruz	Cat#sc-2005; RRID: AB_631736
EV-hCD9-A488	Unchained Labs	Cat#283-034
EV-hCD63-A647	Unchained Labs	Cat#283-014
EV-hCD81-A555	Unchained Labs	Cat#283-024
Mitochondria Antibody (113-1)	Novus	Cat#NBP2-32980; RRID: AB_3282881
Vimentin	R&D systems	Cat#MAB2105; RRID: AB_2241653
CD31 (PECAM-1) Monoclonal Antibody (390)	eBioscience	Cat#14-0311-82; RRID: AB_467201
Anti-CD31 antibody (EPR3094)	Abcam	Cat#ab76533; RRID: AB_1523298
Anti-Ki67 antibody [SP6]	Abcam	Cat#ab16667; RRID: AB_302459
Anti-Cathepsin K antibody	GeneTex	Cat#GTX59712; RRID: AB_10723347
Alexa Fluor 488 Goat anti-Rabbit IgG	Invitrogen	Cat#A-11008; RRID: AB_143165
Alexa Fluor 647 Goat anti-Rabbit IgG	Invitrogen	Cat#A-21244; RRID: AB_2535812
Alexa Fluor 488 Goat anti-rat IgG	BioLegend	Cat#405418; RRID: AB_2563120
Alexa Fluor 647 Goat anti-rat IgG	BioLegend	Cat#405416; RRID: AB_2562967
Alexa Fluor 594 Goat anti-mouse IgG	BioLegend	Cat#405326; RRID: AB_2563308
Goat F(ab) Anti-Mouse IgG H&L	Abcam	Cat#ab6668; RRID: AB_955960

**Bacterial and virus strains**		

Firefly Luciferase Lentivirus	BPS Bioscience	Cat#79692-Puromycin

**Biological samples**		

Human tissues	This study	N/A

**Chemicals, peptides, and recombinant proteins**		

KGM Gold Keratinocyte Growth Medium BulletKit	Lonza	Cat#00192060
KBM Gold Basal Medium	Lonza	Cat#00192151
Keratinocyte SFM	Gibco	Cat#10724011
Keratinocytes Supplements	Gibco	Cat #37000-015
Y-27632	Selleckchem	Cat#S1049
Polybrene	Santa Cruz Biotechnology	Cat#sc-134220
Puromycin	Sigma	Cat#P7255
MEM α (Minimum Essential Medium α)	Gibco	Cat#12561072
Dulbecco’s Modified Eagle Medium/Nutrient Mixture F-12	Gibco	Cat#11330032
Fetal Bovine Serum, Value	Gibco	Cat#A5256701
Antibiotic-Antimycotic (100×) (penicillin, streptomycin, and Gibco Amphotericin B)	Gibco	Cat#15240062
Accutase	Merck	Cat#SCR005
0.05% Trypsin-EDTA	Gibco	Cat#25300062
CELLBANKER 1	ZENOGEN PHARMA	Cat#CB011
NutriFreez D10 Cryopreservation Medium	Sartorius	Cat#05-713-1B
Pierce BCA Protein Assay Kits	Thermo Fisher Scientific	Cat#23225
Clarity Max Western ECL Substrate	BioRad	Cat#1705062
Phalloidin, Alexa Fluor™ 488	Invitrogen	Cat#A12379
DAPI Staining Solution	Abcam	Cat#ab228549
Matrigel	Corning	Cat#354234
CellTracker Red	Invitrogen	Cat#C34552
CellTracker Green	Invitrogen	Cat#C7025
TRIzol	Invitrogen	Cat#15596026
TRIzol LS	Invitrogen	Cat#10296010
Protector RNase inhibitor	Merck	Cat# 3335402001

**Critical commercial assays**		

Molecular-weight-cutoff ultrafiltration, 30 kDa	Cytiva	Cat#GE28-9323-61
Leprechaun Exosome Human Tetraspanin Kit	Unchained Labs	Cat#251-1044
PKH26 Red Fluorescent Cell Linker Kits	Sigma	Cat#MINI26
Chromium Next GEM Single Cell Multiome Reagent Kit A, 4 rxns	10xGenomics	Cat#PN-1000284
Chromium Next GEM Single Cell Multiome ATAC Kit A, 4 rxns	10xGenomics	Cat#PN-1000281
Library Construction Kit B, 4 rxns	10xGenomics	Cat#PN-1000279
Chromium Next GEM Chip J Single Cell Kit, 16 rxns	10xGenomics	Cat#PN-1000230
Single Index Kit N Set A, 96 rxns	10xGenomics	Cat#PN-1000212
Trichrome Stain Kit (Modified Masson’s)	ScyTek Laboratories	Cat#TRM-1

**Deposited data**		

mRNA sequencing	This paper	GEO: GSE317372
MicroRNA sequencing	This paper	GEO: GSE317373
Single-nucleus ATAC and RNA sequencing	This paper	GEO: GSE317374
Analyzed data	This paper	https://doi.org/10.5281/zenodo.18373021

**Experimental models: Cell lines**		

AM-1	Gift from Dr. Hidemitsu Harada	N/A

**Experimental models: Organisms/strains**		

CAnN.Cg-*Foxn1*^*nu*^/CrlNarl mice (nude mice)	National Center for Biomodels	N/A
NOD.Cg-*Prkdc*^*scid*^*Il2rg*^*em1Narl*^/Narl mice (ASID mice)	National Center for Biomodels	N/A

**Software and algorithms**		

IDEAS (Image Data Exploration And Analysis Software)	CYTEK	https://cytekbio.com/pages/imagestream
Imaris Viewer	OXFORD	https://imaris.oxinst.com/imaris-viewer
QuPath	Bankhead, P. et al.^34^	https://qupath.github.io/
ZEISS arivis	ZEISS	https://www.zeiss.com/microscopy/en/products/software/advanced-image-analysis.html
GraphPad Prism (v10)	Dotmatics	https://www.graphpad.com
ImageJ	ImageJ	https://imagej.net/ij
FlowJo (v10)	BD	https://www.flowjo.com
CLC Genomics Workbench	QIAGEN	https://digitalinsights.qiagen.com
Ingenuity Pathway Analysis (IPA)	QIAGEN	https://digitalinsights.qiagen.com
R studio (v4.5.1)	R Foundation	https://www.r-project.org
EnhancedVolcano (1.26.0)	Blighe et al.^35^	https://bioconductor.org/packages/release/bioc/html/EnhancedVolcano.html
clusterProfiler (4.16.0)	Yu et al.^36^	https://bioconductor.org/packages/release/bioc/html/clusterProfiler.html
Fgsea (1.34.2)	Korotkevich et al.^17^	https://bioconductor.org/packages/release/bioc/html/fgsea.html
GseaVis (0.1.1)	Zhang et al.^37^	https://github.com/junjunlab/GseaVis
Seurat (v5)	Hao et al.^38^	https://cran.rstudio.com
Signac (1.16.0)	Stuart et al.^39^	https://cran.rstudio.com
Monocle 3 (1.4.26)	Trapnell et al.^40^	https://github.com/cole-trapnell-lab/monocle3
chromVAR (1.30.1)	Schep et al.^19^	https://bioconductor.org/packages/release/bioc/html/chromVAR.html
JASPAR 2020	Fornes et al.^41^	https://www.bioconductor.org/packages/release/data/annotation/html/JASPAR2020.html

### Experimental model and study participant details

#### Animal handling and ethics

All animal experiments were conducted in accordance with the ethical guidelines of the National Defense Medical University animal care procedures, following protocol number NDMU-24-259 and NDMU-25-100 approved by the Animal Care Committee. All mice were obtained from the National Center for Biomodels, Taiwan. Eight to ten weeks male and female nude mice (CAnN.Cg-*Foxn1*^*nu*^/CrlNarl mice, subcutaneous xenograft) and eight to ten weeks male and female advanced severe immunodeficiency (ASID) mice (NOD.Cg-*Prkdc*^*scid*^*Il2rg*^*em1Narl*^/Narl) (intraosseous orthotopic model) were used. Mice were randomly allocated to experimental groups with equal numbers of each sex. As we did not observe sex differences, male and female mice were analyzed together in all experiments. Weekly body weight checks after tumor transplantation were performed, and mice were euthanized if they lost more than 20% of their body weight or displayed signs of distress. Euthanasia at indicated tissue-collection time points was performed using carbon dioxide asphyxiation followed by cervical dislocation. Animals were maintained under controlled housing conditions with regulated temperature, humidity, and light–dark cycles. Animal welfare was assessed daily by trained personnel throughout the experimental period. Appropriate measures were taken to minimize unnecessary discomfort and distress, and any signs of illness or abnormal behavior were promptly identified and managed according to institutional animal care guidelines.

#### Tissue collection

The collection and use of ameloblastoma specimens were conducted under a protocol approved by the Institutional Review Board of Tri-Service General Hospital (B202105164), including specimens obtained through participating institutions. Fresh ameloblastoma (AM) and matched normal gingival tissue samples were obtained post-surgery. These included one conventional AM (CONV01, follicular/plexiform) from Chung Shan Medical University Hospital, and five samples from Tri-Service General Hospital: two unicystic mural AM (UCm01 and UCm02, plexiform) and three conventional AM (CONV02, desmoplastic; CONV03 and CONV04, follicular/plexiform). Two control normal gingiva tissues from healthy subjects, as well as dental follicle and dental pulp tissues obtained from the same healthy subject following impacted third molar extraction, were collected at the Department of Oral and Maxillofacial Surgery at Tri-Service General Hospital (IRB number: A202105108). Informed consents were obtained from all patients. Two independent board-certified oral pathologists reviewed the histopathological and radiographic features of each case and confirmed the diagnoses according to the diagnostic criteria for odontogenic tumors described in the World Health Organization Classification of Tumours, Head and Neck Tumours, 5th edition.^42^^,^^43^ Conventional AM was diagnosed based on characteristic histopathological features, including follicular, plexiform, or desmoplastic patterns, together with corresponding radiographic findings, such as multilocular radiolucent lesions. The follicular pattern resembles the epithelial component of the enamel organ within a fibrous stroma and is characterized by peripheral palisading ameloblast-like cells with reverse nuclear polarity and central stellate reticulum-like cells. The plexiform pattern consists of anastomosing strands of ameloblastomatous epithelium with inconspicuous stellate reticulum and cyst-like stromal degeneration. The desmoplastic pattern is characterized by a densely collagenous stroma. Unicystic AM was diagnosed based on a well-defined unilocular radiolucent lesion and a cystic cavity lined by ameloblastomatous epithelium. The mural subtype was defined by ameloblastomatous epithelial proliferation extending into the cyst wall. The age, sex, diagnosis, histological subtype, tissue source, and derived primary cell types for each donor are provided in Tables S1 and S2. Gender, race, ethnicity, and ancestry were not collected/reported in this study.

#### Cell line and luciferase lentivirus transduction

The human ameloblastoma cell line, AM-1, was generously provided by Dr. Hidemitsu Harada at Iwate Medical University. The AM-1 cell line was subjected to short tandem repeat (STR) profiling by GenLabs (Taiwan) using the GenePrint 24 System (Promega). The obtained STR profile showed no ≥ 80% match with other human cell lines in the reference databases examined. AM-1 cells were cultured with defined serum-free KGM Gold Keratinocyte Growth Medium BulletKit (KGM Gold, Lonza, #00192060). AM-1 cells were cultured in the 24-well plate until reaching approximately 70–80% confluent, followed by transduction with Firefly Luciferase Lentivirus particles (BPS Bioscience, #79692-Puromycin) in the presence of 5 μg/mL Polybrene (Santa Cruz Biotechnology, #sc-134220) to increase the infection efficiency. After transduction for 10 hours, the viral particle-containing medium was removed and replenished with the fresh KGM Gold medium. After stably cultured for 1-2 days, puromycin (1, 2, 4, 6, 8, and 10 μg/ml) was introduced into the culture to select the optimal concentration of puromycin that can be tolerated by positively transduced cells. AM-1 cells that were successfully transduced with Luciferase Lentivirus (AM-1^luc^) were selected and continuously expanded in the presence of 1μg/ml of puromycin. AM-1 cells and AM-1^luc^ cells were cryopreserved in serum containing cryopreservation medium (CELLBANKER 1). AM-1 and AM-1^luc^ cells were also tested for mycoplasma contamination and were negative. All the procedures were conducted under a Biosafety Level 2 laboratory.

#### Isolation of primary cells

Primary ameloblastoma-derived epithelial cells (AMEpiCs), ameloblastoma-derived mesenchymal stromal cells (AMMSCs), gingival-derived MSCs (GMSCs), dental follicle-derived MSCs (DFMSCs) and dental pulp-derived MSCs (DPMSCs) were isolated as previously described.^16^^,^^44^^,^^45^^,^^46^ Briefly, dental pulp tissues were obtained from root fragments sectioned during complicated odontotomy procedures. All fresh tissues (at least 3-6 mm^3^) were minced into 0.5-1 mm^3^ pieces, followed by enzymatic digestion with 0.2% collagenase type I for 1 hour in a 37 °C shaking incubator. AM tumors were carefully dissected from the bone, and only solid tumor tissues were collected to avoid the inclusion of bony structures.

AMEpiCs and AMMSCs were isolated from dissociated AM tumor tissues using medium selection. For AMEpiCs, the dissociated tumor cells were seeded in tissue culture dishes (2x10^4^ cells/cm^2^) in defined serum-free KGM Gold with 10μM Y-27632. For AMMSCs, the dissociated tumor cells were seeded in complete α-MEM medium (α-MEM with 10% FBS and 1% penicillin/streptomycin/amphotericin B). For other normal MSCs, dissociated cells were seeded in the same complete α-MEM medium. All cells were maintained at 37 °C in a humidified incubator with 5% CO_2_. After 48 hours, the non-adherent cells were removed, and fresh media were replenished every three days.

When cells were at 75%-95% confluence, AMEpiCs were passaged using Accutase (Merck, #SCR005), while MSCs were passaged using 0.05% Trypsin-EDTA solution (Gibco, #25300062). For MSC passaging, a short dissociation time of approximately 2 minutes was used to preferentially detach stromal cells while retaining epithelial islands, which typically required 6-10 minutes to detach from the culture dishes. Primary AMEpiCs at early passages were cryopreserved in CELLBANKER, MSCs at early passages were cryopreserved in NutriFreez D10 Cryopreservation Medium, and only cells at fewer than six passages were used for subsequent experiments. Donor information, including age, sex, diagnosis, tissue source, and primary cell types derived from each specimen, is summarized in Tables S1 and S2 arized in Tables S1 and S2.

#### Isolation of mesenchymal stromal cell-derived secretome

Mesenchymal stromal cells (MSCs) were cultured in complete α-MEM until reaching approximately 80% confluence. Then, cells were washed with PBS twice and cultured in serum-free α-MEM for 48 hours (10 mL per 10-cm dish). The conditioned medium was subsequently collected and centrifuged at 4,400 rpm for 20 min to remove cellular debris. The supernatant was then concentrated using a 30 kDa molecular-weight-cutoff ultrafiltration unit (Cytiva, #GE28-9323-61) by centrifugation at 4,400 rpm for approximately 30 min, repeated twice, with one intermediate wash using PBS. This concentration yielded 100x-concentrated MSC-derived secretome, which was used for downstream experiments. The total protein concentration of the secretome was quantified using Pierce BCA Protein Assay Kits (Thermo Fisher Scientific, #23225). To estimate the approximate number of AMMSCs corresponding to each secretome treatment concentration, the total number of AMMSCs used for conditioned medium collection was normalized to the total protein yield of the concentrated secretome. Based on this calculation, 10 μg/mL secretome corresponded approximately to the secretome collected from 1.5x 10^5^ AMMSCs per treatment unit (1 mL medium), whereas 20 μg/mL corresponded approximately to the secretome collected from 3x10^5^ AMMSCs per treatment unit (1 mL medium). The estimated cell number was calculated using the following formula: estimated cell number per treatment unit = total AMMSC number x treatment protein (μg) / total concentrated secretome protein (μg).

### Method details

#### Western blot

Cells were lysed with RIPA buffer (with protease and phosphatase inhibitors), followed by sonication and centrifugation (12,000xg, 10 min, 4 °C). Total protein concentrations were determined using Pierce BCA Protein Assay Kits. Then, 30 μg of total protein was subjected to 10 % SDS-polyacrylamide gels and transferred onto 0.2 μm nitrocellulose membranes (Bio-Rad, Hercules, CA, USA). Membranes were incubated overnight at 4°C with primary antibodies, including LGR5 (Invitrogen, #MA5-25644), non-phospho (Active) β-catenin (Cell Signaling Technology, #8814), fibronectin (Sigma, #F3648), ALDH1 (BD biosciences, #611194), YAP (Cell Signaling Technology, #14074), E-Cadherin (Cell Signaling Technology, #3195), CD63 (Invitrogen, #10628D), CD9 (Invitrogen, #10626D), CD81 (Invitrogen, #10630D), β-actin (Santa Cruz, #sc-47778), followed by horseradish peroxidase-conjugated secondary antibodies for 1 hour. Protein bands were visualized using Clarity Max Western ECL Substrate (BioRad,1705062) and the touch imager (e-Blot, Shanghai, China).

#### Nanoparticle tracking analysis

The MSC-derived secretome was passed through a 0.22 μm membrane filter and analyzed using a NanoSight NS300 (Malvern Panalytical) for nanoparticle size distribution and concentration. A digital sCMOS (scientific Complementary Metal-Oxide-Semiconductor) camera and 488nm laser were used to capture the light scattered by the particles and track motion frame by frame. All analyses were conducted at the Instrument Center, National Defense Medical University.

#### Scanning electron microscopy

MSC-derived secretome was fixed in 2% paraformaldehyde for 15 min and then diluted 1:1 with PBS. A 5 μL aliquot was deposited onto silicon wafers and allowed to air-dry, followed by sputtering a thin layer of gold onto the wafer surface. The sample was then mounted on the SEM stub with an adhesive carbon tape and imaged with a scanning electron microscope (SEM, Thermo Scientific Apreo2s). The image was measured using secondary electron detection with an acceleration voltage of 5kV. All analyses were conducted at the Instrument Center, National Defense Medical University.

#### Image flow cytometry

MSC-derived secretome was labeled with CF Dye (Leprechaun Exosome Human Tetraspanin Kit, Unchained Labs).^47^ All buffers were filtered with 0.22 μm filters prior to use. 100 μL of secretome suspension at 1x10^9^ particles/mL was mixed with 0.6 μL CF Dye stock solution and supplemented with 100 μL of 5% BCA buffer. The mixture was gently mixed and incubated on ice overnight. The following day, samples were centrifuged at 120,000xg for 1.5 hours at 4°C. After centrifugation, the pellet was resuspended in 50 μL of double-distilled water. Then, the samples were further stained with fluorophore-conjugated antibodies against the extracellular vesicle (EV) markers CD9 (EV-hCD9-A488, Unchained Labs, #283-034), CD63 (EV-hCD63-A647, Unchained Labs, #283-014), and CD81 (EV-hCD81-A555, Unchained Labs, #283-024), and analyzed using an imaging flow cytometer (Amnis ImageStreamX Mk II, Luminex Corporation, USA).^48^ Image acquisition and quantitative analyses were performed using IDEAS (Image Data Exploration And Analysis Software). All experiments were conducted at the Instrument Center of the National Defense Medical University.

#### Functional uptake assay

For extracellular vesicle labelling, MSC-derived secretome (20 μg/mL, defined as 1 unit) was first mixed with 500 μL Diluent C and 3 μL PKH26 dye (Sigma, #MINI26), followed by incubation at room temperature for 5 min. After incubation, 10% BSA was added to adjust the volume to 1 mL. Next, the mixture was ultracentrifuged at 190,000 x g for 2 h at 4 °C. Finally, pellets were resuspended in 10 μL PBS per unit and used immediately.

To prepare for uptake experiments, AM-1 cells were seeded at 4x10^4^ cells per well on 0.2% gelatin-coated 8-well chamber slides and cultured in KGM Gold for 3 days. After this period, the medium was replaced with growth factor-free KBM Gold Basal Medium (KBM Gold, Lonza, #00192151). Subsequently, PKH26-labeled MSC-derived secretome (20 μg/mL) was added for 0, 2, 4, and 8 hours, respectively.

After treatment, the AM-1 cells were first washed with PBS, then permeabilized with 0.1% Triton X-100 for 10 min, and subsequently blocked with 2.5% normal goat serum. The cells were stained with phalloidin (1:200; Invitrogen, A12379) for 15 min at room temperature in the dark. Finally, nuclei were counterstained with DAPI Staining Solution (Abcam, #ab228549) and images were acquired using the THUNDER Imager Live Cell (Leica) and processed with Imaris Viewer.

#### Sphere formation

3D-spheroid formation assay was performed as described previously.^16^ Briefly, AM-1 cells or AM-1 cell + AMMSCs (1:1), or AM-1 + AMMSC-derived secretome were seeded at a density of 10^4^ cells/well into Ultralow attached 24-well plates (Corning) with growth factor-free KBM Gold (*n*=3) and cultured for 10 days. Spheres larger than 25 μm were counted, and their sizes and numbers were quantified using GraphPad Prism.

#### Wound healing assay

5x10^5^ AM-1 cells were seeded into a 6-well plate and cultured in supplemented Keratinocyte SFM (Keratinocyte SFM, Gibco, #10724011; Keratinocytes Supplements, Gibco, #37000-015) for about 3 days. After reaching confluence, the cells were washed with PBS and incubated in Keratinocyte SFM without supplements for 2-4 h for starvation. A scratch wound was then generated using a sterile 200-L pipette tip, and the cells were treated with AMMSC-derived secretome (10, 20 L/mL) or PBS (vehicle). Images were acquired at 0, 24, 48, and 72, and the wound area was quantified using ImageJ.^49^

#### Holotomography study

AM-1 cells (110^4^ cells in 50 μL KGM gold) were seeded as a dome at the center of a 35mm glass-bottom dish and allowed to attach for 6 h. The dish was then filled with KGM Gold to a final volume of 1 mL and cultured for 2 days. After stable attachment, the medium was replaced with growth factor-free KBM Gold, and cells were imaged using a low-coherence holotomography (HT) system (HT-X1, Tomocube Inc., Korea), as previously described.^50^^,^^51^ Regions of interest were defined (*n* = 2 per group). For wound healing assay, a scratch was generated using a 10-μL pipette tip. Cells were then treated with PKH-labeled MSC-derived secretome (20 μg/mL), time-lapse imaging was acquired using a 450-nm LED illumination module integrated with a digital micromirror device (DMD) every 20 min for 48 h. An integrated stage top incubation chamber ensures stable physiological conditions of temperature, humidity, and CO2 concentration during long-term imaging over several weeks. All motorized microscopic operations were controlled and monitored by the TomoStudio X operating software (Tomocube Inc., Korea).

#### Flow cytometry

AM epithelial cells (AMEpiCs) or AM-1 cells (1x10^5^ cells per well, 12-well plates) were treated with AMMSC-derived secretome or vehicle for 3 days. Cells were harvested using Accutase, resuspended in cell staining buffer (0.5% BSA in PBS containing 2 mM EDTA), and incubated with a primary antibody against LGR5 (1:100, Invitrogen, #PA5-35304) at 4°C for 30 min. After washing with PBS, cells were incubated with Alexa Fluor 488 goat anti-rabbit secondary antibody (1:200, Invitrogen, #A-11008) in the dark at 4°C for 30 min. Alternatively, cells were directly stained with an APC-conjugated anti-LGR5 antibody (1:100; human, R&D Systems, #FAB8078A) in the dark at 4 °C for 30 min. Samples were analyzed using a BD FACSCalibur or BD Accuri C6 flow cytometer, and data were analyzed with FlowJo software (v10).

#### Spheroid formation in Matrigel

Single-cell suspensions of AM-1 cells, or AM-1 cells + AMMSCs (1:1), were directly dispersed into Matrigel (Corning, #354234) at a density of 1×10^4^ cells/μL (∼40μL each group) and seeded in a dome shape. The dish was inverted during Matrigel solidification to prevent cells from attaching to the culture dish. After solidifying for 20 minutes, the mixture of the cells and Matrigel was cultured in 50% KGM Gold and 50% Dulbecco’s Modified Eagle Medium/Nutrient Mixture F-12 (DMEM/F-12, Gibco, #11330032). Spheroid formation in Matrigel was observed under a microscope every 2-3 days, and the entire Matrigel-containing spheroids culture was harvested on day 10.

For experiments requiring inverted multiphoton confocal imaging, cells (2x10^5^) were counted and transferred to 1.5mL microcentrifuge tubes. AM-1 cells were stained with CellTracker Red (Invitrogen, #C34552), and AMMSCs were stained with CellTracker Green (Invitrogen, #C7025), each at a dilution of 1:1000, and incubated at 37 °C for 15 minutes. Cells were centrifuged at 1,300 rpm for 5 min at 4 °C, washed with PBS, and then resuspended in 40 μL Matrigel. The cell-Matrigel mixture was carefully seeded onto prewarmed 35mm glass-bottom dishes and solidified at 37 °C for 20minutes. Culture medium was then added to fully cover the Matrigel. Three-dimensional images were acquired on days 0 and 10 using an inverted multiphoton confocal microscope (Zeiss LSM 980).

To prepare the Matrigel for frozen section, the gel was washed twice with PBS, and the entire Matrigel, including organoids, was fixed in 4% PFA for 15 minutes, followed by washing with PBS twice for 15 minutes each. The spheroids with Matrigel were detached from the dish with a fine flat spatula and transferred to the mold. The entire Matrigel-containing spheroid culture was embedded in OCT for frozen section. Both H & E staining and immunofluorescence studies on the expression of CK14 (Invitrogen, #MA5-11599) and fibronectin were performed.

#### Subcutaneous xenograft

Eight-week-old female and male CAnN.Cg-*Foxn1*^*nu*^/CrlNarl (nude) mice were purchased from the National Center for Biomodels, Taiwan. All animal procedures were handled according to the guidelines of the Institutional Animal Care and Use Committee (IACUC) at the National Defense Medicine University. Three group spheroids in Matrigel were cultured in KBM Gold for 3 days: (1) 8x10^5^ AM-1 cells alone; (2) 8x10^5^ AM-1 cells co-cultured with AMMSCs (1:1); and (3) 8x10^5^ AM-1 cells treated with AMMSC-derived secretome (20 μg/mL). Nude mice were randomly assigned to three groups (*n* = 4 per group), and tumor spheroids were subcutaneously transplanted into the dorsal skin. Xenografted tumors were harvested 4 weeks after transplantation for histological analysis.

#### mRNA sequencing and gene set enrichment analysis

AM-1 cells (3x10^5^ per well) were seeded in 6-well plates and cultured in KGM Gold for 2 days, followed by replacement with KBM Gold and treatment with AMMSC-, paired GMSC-derived secretome or vehicle (*n* = 3 per group; CONV01-03). After 72 h, cells were lysed in TRIzol (Invitrogen, #15596026) and stored at -80 °C prior to RNA isolation, library preparation, and sequencing at Azenta Life Sciences (USA).

Differentially expressed genes were analyzed using CLC Genomics Workbench (QIAGEN), and volcano plots were generated in R (version 4.5.1) with Bioconductor (version 3.21) using the EnhancedVolcano R package^35^ (*p* < 0.05, |log_2_ fold change| > 1). Gene set enrichment analysis (GSEA) was performed using the clusterProfiler^36^^,^^52^ and fgsea^17^ packages. The curated gene sets (C2) were obtained from the Molecular Signatures Database (MSigDB). Gene sets with adjusted *p*-values <0.05 were considered significant. Enriched pathways were summarized by normalized enrichment score (NES) and visualized using dot plots generated with the GseaVis package.^37^ Representative pathways were further illustrated using _GSE_A enrichment plots to display running enrichment scores and ranked gene distributions.

#### MicroRNA seq and analysis

AMMSC- and GMMSC-derived secretomes were mixed with TRIzol LS reagent (1:3, v/v) and stored at -80 °C prior to small RNA isolation, library preparation, and microRNA sequencing performed by Welgene Biotech (Taiwan). Differentially expressed microRNAs were visualized using volcano plots generated with the EnhancedVolcano R package^35^ (*p* < 0.05, |log_2_ fold change| > 0.58). Integration of microRNA and mRNA expression data was performed using QIAGEN Ingenuity Pathway Analysis (IPA) network analysis.^53^

#### Single-nucleus ATAC and RNA sequencing and analysis

Primary AMEpiCs (8x10^5^) were seeded in 6-cm dishes and cultured in KGM Gold for 2 days, followed by treatment with paired AMMSC-derived secretome (CONV02) and cultured in KBM Gold for 72 hours. Nuclei isolation was followed by 10x Genomics protocol (CG000365). Chilled lysis buffer: 10 mM Tris-HCl (pH 7.4), 10 mM NaCl, 3 mM MgCl_2_, 0.1% Tween-20, 0.1% Nonidet P40 Substitute, 0.01% Digitonin, 1% BSA, 1mM DTT, and 1 U/μl RNase inhibitor, and wash buffer: 10 mM Tris-HCl (pH 7.4), 10 mM NaCl, 3 mM MgCl_2_, 1% BSA, 0.1% Tween-20, 1mM DTT, and 1 U/μl RNase inhibitor, were prepared in nuclease-free water and used immediately. AMEpiCs were dissociated using Accutase, transferred to a 1.5 mL microcentrifuge tube, and washed with 0.04% BSA in PBS twice by centrifuging at 300xg for 5 min at 4°C. The cell pellet was resuspended in chilled lysis buffer (100 μL), pipetted 10 times, and incubated for 3 min on ice. 1 mL chilled wash buffer was added to the lysed cells, the mixture was pipetted 5 times, centrifuged at 500xg for 5 min at 4°C, and the procedure was repeated 2 more times. The nuclei were resuspended in 1X Nuclei Buffer (10x Genomics) with 1mM DTT and 1 U/μl RNase inhibitor, and nuclei quality was confirmed microscopically.

We aimed to recover 5000 nuclei and processed the sample according to the Chromium Next GEM Single Cell Multiome ATAC + Gene Expression User Guide (CG000338). Briefly, the nuclear suspension was incubated in a Transposition Mix containing a Transposase to fragment the DNA at open chromatin regions and add adapter sequences. The samples and 10x Barcoded Gel Beads were then loaded onto a Chromium Next GEM Chip J to generate Gel Beads-in-emulsion (GEMs), allowing for the barcoding of transposed DNA and the reverse transcription of poly-adenylated mRNA into cDNA. Then, pre-amplification PCR, ATAC library construction, cDNA amplification, and gene expression library construction were performed at the Instrument Center of the National Defense Medical University, followed by sequencing on an Illumina NovaSeq 6000 sequencer with a sequencing depth of at least 25k reads for ATAC-seq and 20K reads for RNA-seq (performed by Genomics BioSci & Tech Co., Ltd., Taiwan). Raw data were aligned to the GRCh38 reference genome and processed using Cell Ranger ARC (10x Genomics).

Analyses were performed in R (version 4.5.1) with Bioconductor (3.21) using the R packages Seurat (v5)^38^ and Signac^39^ (version 1.16.0), with weighted nearest neighbor (WNN) analysis to integrate snATAC-seq and snRNA-seq data.^18^ Pseudotime trajectories were inferred using Monocle 3^40^^,^^54^ (version 1.4.26), and TF activity was calculated using chromVAR^19^^,^^55^ (version 1.30.1) against the JASPAR 2020 motif database.^41^

#### Establishment of orthotopic mouse model of human ameloblastoma

Ten-week-old female and male advanced severe immunodeficiency (ASID) mice (NOD.Cg-*Prkdc*^*scid*^*Il2rg*^*em1Narl*^/Narl) were purchased from the National Center for Biomodels, Taiwan. ASID mice are extremely deficient in NK cells, natural killer T cells, T cells, and B cells. All animal procedures were performed according to the guidelines of the Institutional Animal Care and Use Committee of IACUC at the National Defense Medicine University. Before transplantation, three types of tumor spheroids were generated using AM-1^luc^ cells: (1) AM-1^luc^ only (1.2 x 10^6^ cells per group); (2) AM-1^luc^ treated with AMMSC-derived secretome (1.2 x 10^6^ cells per group); and (3) AM-1^luc^ combined AMMSCs (1:1 ratio, 1.2 x 10^6^ cells per group), whereby cells were cultured in a 6-well Ultra-Low Attachment plate (Corning) using KGM Gold medium for three days. We randomly transplanted spheroids from three groups into one site of mouse maxilla. Briefly, an envelope gingival flap was reflected to expose the maxillary ridge in front of the upper first molar, and a bony cavity (about 0.3mm X 0.2 mm X 0.15 mm) was prepared in front of the first molar by surgical burs (Figures 4B and 4C). Then, a solidified mixture of cell spheroids and 4μL Matrigel was transplanted into the bony cavity, and the wound was primarily closed with 7-0 silk and sealed with Vetbond Tissue Adhesive (3M, #1469SB) to improve the soft tissue healing (Figures 4D and 4E). The sham control was performed by implanting solidified Matrigel (4μL) into the bony cavity. The surgical procedures were performed under loupes (SurgiTel UltiView Pro Loupes, magnification powers of 5.5x).

#### Immunofluorescence study

The samples were fixed in 4% paraformaldehyde (Santa Cruz) for at least 48 hours at 4 °C and the mouse maxilla was decalcified in 20% EDTA solution for about three weeks at 4°C with intermittent shaking. Then, the samples were embedded in either paraffin or optimal cutting temperature (OCT) compound. For dual-color Immunofluorescence (IF) study, frozen sections were permeabilized in 0.5% triton X-100 in PBS for 15 minutes and then blocked in 10% goat serum at room temperature for 1 hour. For mouse sample, we utilized Goat F(ab) Anti-Mouse IgG H&L (1:1000, Abcam, #ab6668) at room temperature for one hour for mouse on mouse blocking, and all sample followed by incubation at 4 °C overnight with a primary antibody for mouse anti-human mitochondria (Novus, #NBP2-32980), and rat anti-vimentin (R&D, #MAB2105) in combination with rabbit-derived primary antibodies, including CD31(rabbit anti-human: Abcam, #ab76533; rat anti-mouse: eBioscience, # 14-0311-82), LGR5 (OriGene, #TA324730), non-phospho (Active) β-catenin (Cell Signaling, #8814), fibronectin (Sigma, #F3648), Ki-67 (Abcam, #ab16667) and Cathepsin K (GeneTex, #GTX59712). Afterward, the sections were incubated at room temperature for 1 h with appropriate fluorochrome-conjugated secondary antibodies: Alexa Fluor 488 Goat anti-rabbit IgG, Alexa Fluor 647 Goat anti-rabbit IgG, Alexa Fluor 488 Goat anti-rat IgG, Alexa Fluor 647 Goat anti-rat IgG, Alexa Fluor 594 Goat anti-mouse IgG. Isotype-matched control antibodies were used as negative controls. Nuclei were counterstained with DAPI Staining Solution and images were acquired using the THUNDER Imager Live Cell (Leica) and processed with Imaris Viewer. Mean fluorescence intensity (MFI) was quantified using ImageJ. Ki-67 positive percentage and nuclear β-catenin colocalization were quantified using QuPath.^34^ Tumor epithelial islands were manually annotated as regions of interest (ROIs) based on h-mitochondria-positive tumor epithelial islands. Cells were detected using the DAPI channel. Ki-67-positive nuclei and nuclear β-catenin colocalization were identified based on cell nuclei Ki-67 or β-catenin fluorescence using the same threshold across all experimental groups.

#### Modified Masson’s trichrome staining

Frozen sections were stained using a Trichrome Stain Kit (Modified Masson’s, ScyTek Laboratories) according to the manufacturer’s instructions. Briefly, sections were rehydrated to distilled water and incubated in preheated Bouin’s fluid (60 °C) for 60 min, followed by rinsing in tap water and rehydration to distilled water. Sections were then stained with working Weigert’s iron hematoxylin for 8 min, rinsed in tap water, and rehydrated to distilled water. This was followed by staining with Biebrich scarlet/acid fuchsin solution for 15 min and rinsing in distilled water. Sections were differentiated in phosphomolybdic/phosphotungstic acid solution for approximately 15 min until collagen staining (red) was cleared. Without rinsing, sections were stained with aniline blue solution for 5 min, rinsed in distilled water, and treated with 1% acetic acid for 5 min. Sections were then rapidly dehydrated through two changes of 95% ethanol and two changes of absolute ethanol, cleared in xylene, mounted, and scanned using a ZEISS Axioscan 7 digital slide scanner.

#### *In vivo* bioluminescence imaging and three-dimensional micro-computed tomography

Tumor growth was tracked using a non-invasive 3D *In Vivo* Imaging System (IVIS Spectrum, PerkinElmer) on Day 1, Day 14, Day30, Day 60 and Day 90, and live micro-computed tomography (Quantum GX2 Micro CT, PerkinElmer) on Day 1, Day 60 and Day 90. The co-registration images of 3D bioluminescence and micro-CT images were conducted with Living Image 2.50 software. The mice were sacrificed after three and four months and the intraoral photos were captured using a digital microscope (Dino-Lite Edge 3.0). Micro-CT images were reconstructed with Materialise Mimics and 3D Slicer software. The tumor volume was measured using ZEISS arivis. Briefly, micro-CT DICOM datasets of mouse jaw tumors were imported into ZEISS arivis software and enhanced using a Richardson-Lucy deconvolution filter to improve visualization of tumor boundaries. Tumor regions were manually segmented slice by slice, and the segmented tumor volume was used for quantitative comparison among experimental groups.

### Quantification and statistical analysis

#### Statistical analysis

Data were analyzed using one-way ANOVA followed by Dunnett’s or Tukey’s multiple comparisons test, or by two-way ANOVA/mixed-effects modeling followed by Tukey’s multiple comparisons test, as appropriate. Statistical analyses were performed using GraphPad Prism 10. *p* values less than 0.05 were considered statistically significant.
