# Supplementary material for: Stroma-oriented tumor microenvironment facilitates intraosseous ameloblastoma tumor growth
Source: iScience. 2026 Aug 27;29(9):117344. doi: 10.1016/j.isci.2026.117344 (PMC13546885; doi:10.1016/j.isci.2026.117344)
Supplement: Supplementary file 1 [file mmc1.pdf]

iScience, Volume *29*

Supplemental information

Stroma-oriented tumor microenvironment facilitates intraosseous ameloblastoma tumor growth

Da-Yo Yuh, Chi-Ju Yao, Chia-Yu Chen, Ching-Yi Wu, Jian-Liang Chou, Hui-Na Chou, Ling-Yu Kung, Yi-Tzu Chen, Wei-Chin Chang, Yuan-Wu Chen, Qunzhou Zhang, Anh D. Le, and Ting-Han Chang

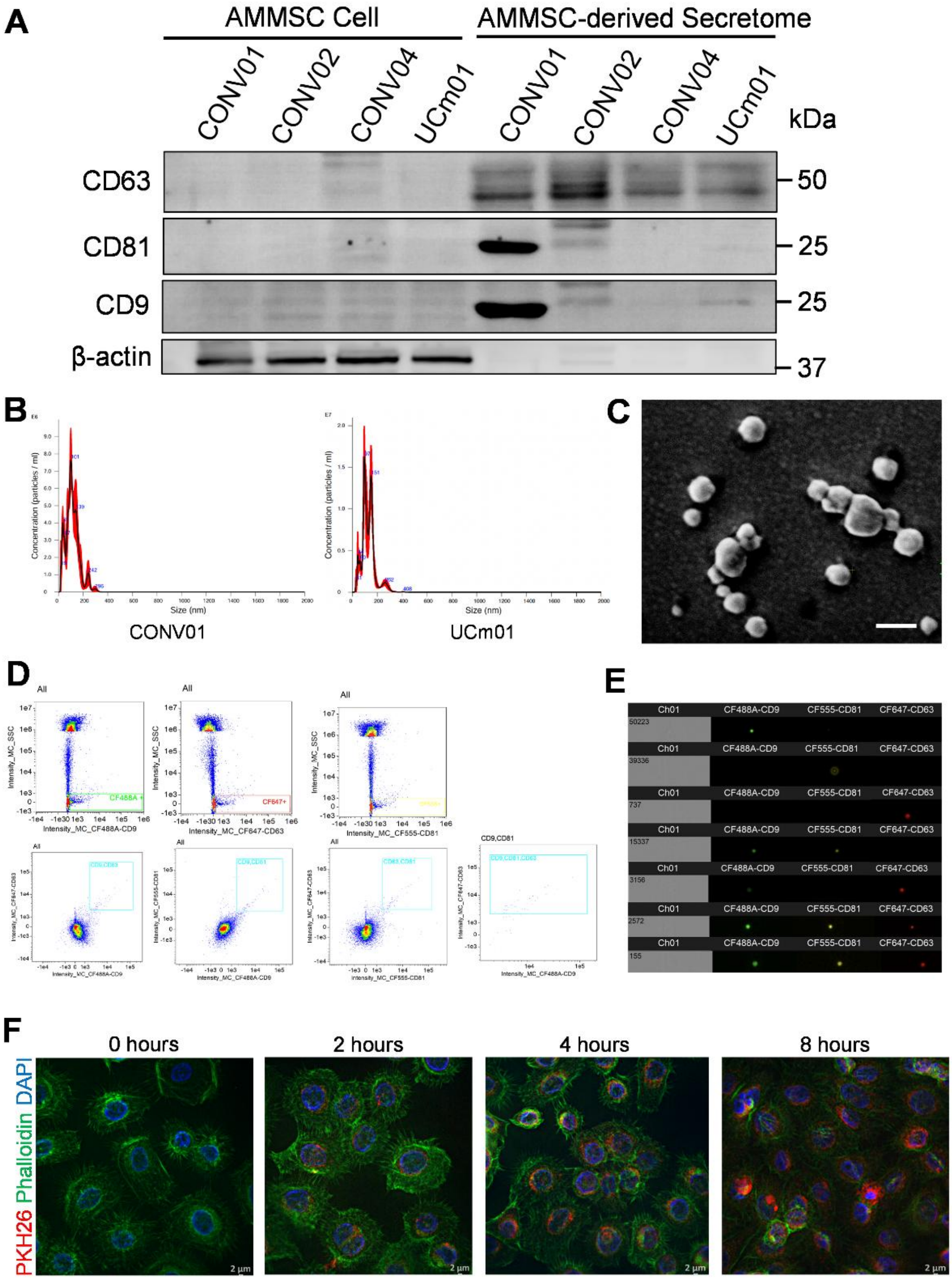

Figure S1. Characterization of extracellular vesicles in AMMSC-derived secretome. (A) Western blot analysis showing expression of the extracellular vesicle (EV) marker CD63 in secretomes derived from primary AMMSCs from four patients; β-actin was detected in corresponding cell lysates as a cellular control. Three independent experiments. (B) AMMSC-derived secretome was filtered through a 0.22-μm membrane, followed by nanoparticle tracking analysis (NTA) to determine the size of small EVs. Two independent experiments. (C) Scanning electron microscopy (SEM) revealed round EV-like structures with diameters of approximately 100 nm in unfiltered AMMSC-derived secretome. Scale bar, 100 nm. Two independent experiments. (D and E) Imaging flow cytometry analysis showing EVs labeled with CD63 (CF647), CD9 (CF488), and CD81 (CF555), either as single-positive or co-expressing populations, in AMMSC-derived secretome. Two independent experiments. (F) Uptake assay demonstrating internalization of PKH26-labeled EVs from AMMSC-derived secretome into AM-1 cells. Scale bars, 2 μm. Three independent experiments. Related to Figure 1.

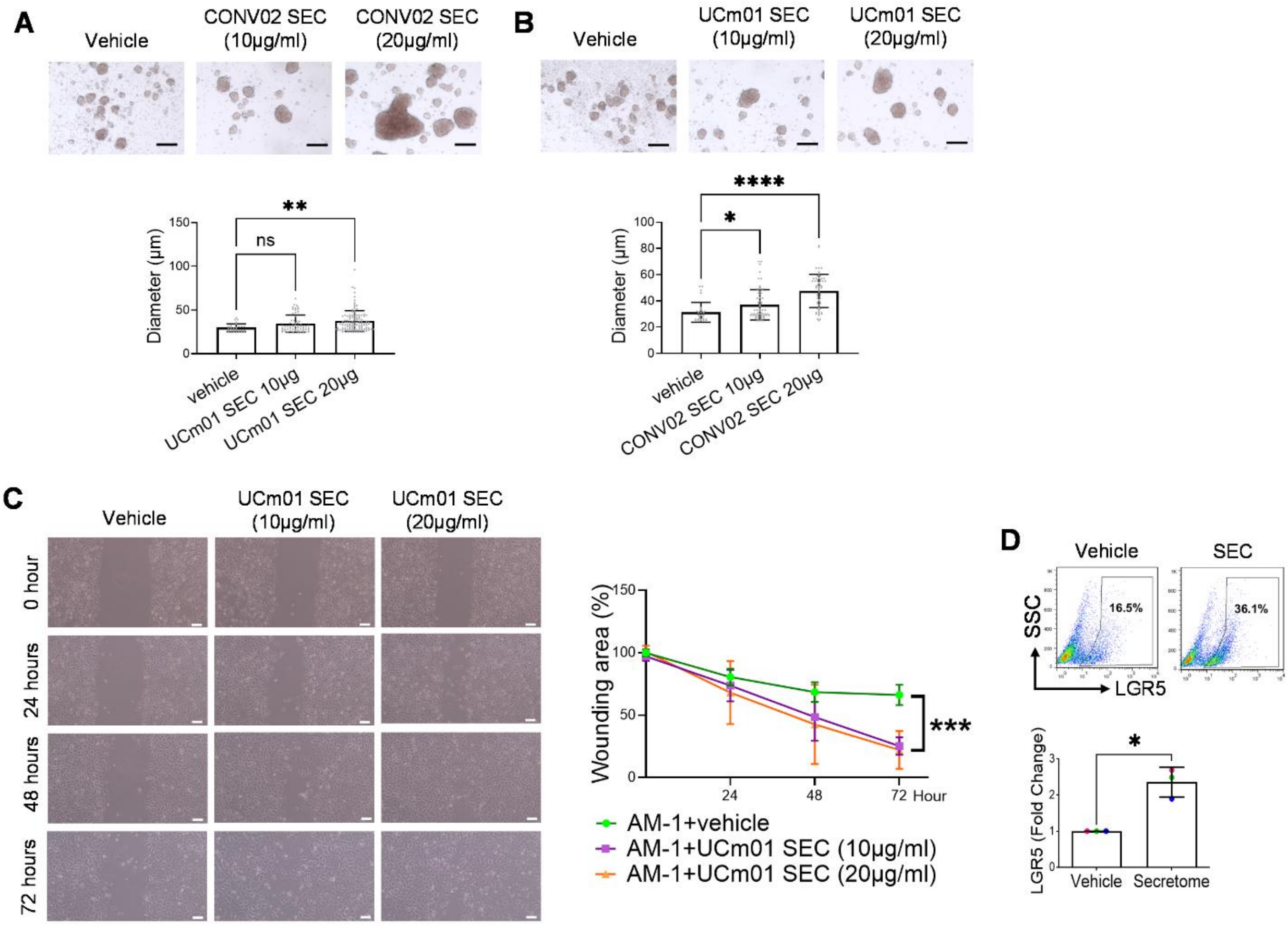

Figure S2. AMMSC-derived secretome enhances 3D-sphere formation and migration. (A) Representative images of sphere formation of AM-1 cells combined with AMMSC CONV02-derived secretome (SEC) for 10 days. Scale bars, 100 µm. Data are mean ± SD. One-way ANOVA, Tukey's multiple comparisons test. n=3 independent experiments from CONV02. ns= not significant, ***p*<0.01. (B) Sphere formation of AM-1 cells combined with AMMSC UCm01-derived secretome (SEC) for 10 days. Scale bars, 100 µm. Data are mean ± SD. One-way ANOVA, Tukey's multiple comparisons test. n=3 independent experiments from UCm01. **p*<0.05, *****p*<0.0001. (C) Wound healing assay of migrating AM-1 cells treated with AMMSC UCm01-derived secretome. Scale bars, 30 µm. Data are mean ± SD. Two-way ANOVA, Tukey's multiple comparisons test. n=4 independent experiments from UCm01. ****p*<0.001. (D) Flow cytometry analysis showing increased LGR5 expression in AM-1 cells treated with AMMSC-derived secretome (SEC, 10µg/mL) for 3 days. Upper, representative dot plots; lower, quantitative analysis. Data are mean ± SD. Paired t test, n=3 independent AMMSC-derived secretome (CONV01, CONV02 and CONV03). *p<0.05. Related to Figure 1.

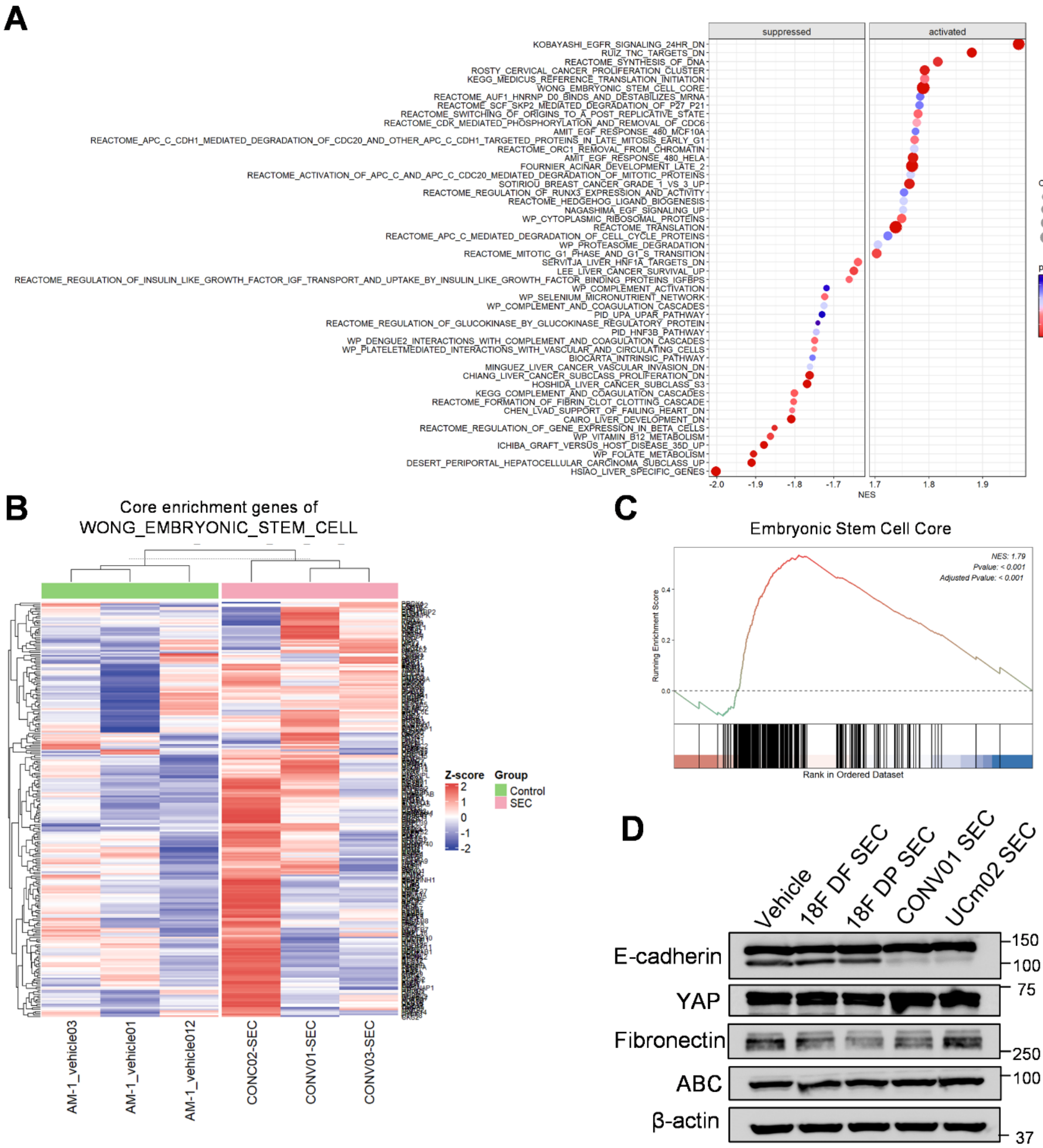

Figure S3. AMMSC-derived secretome promotes stemness- and epithelial-mesenchymal transition-related gene expression. AM-1 cells treated with AMMSC-derived secretome or vehicle for 3 days. (A-C) mRNA sequencing analysis. Three biological replicates. (A) Normalized enrichment scores (NES) of hallmark pathways derived from gene set enrichment analysis (GSEA). (B) Heatmap showing expression of genes in the WONG EMBRYONIC STEM CELL gene set. (C) Representative GSEA enrichment plots. (D) Western blot analysis showing that AMMSC-derived secretome (SEC, 20μg/mL, CONV01 SEC and UCm02 SEC) increased fibronectin and decreased E-cadherin expression in AM-1 cells compared with secretomes (SEC, 20μg/mL) derived from dental follicle MSCs (DFMSCs) or dental pulp MSCs (DPMSCs). Two independent experiments. Related to Figure 1 and Figure 2.

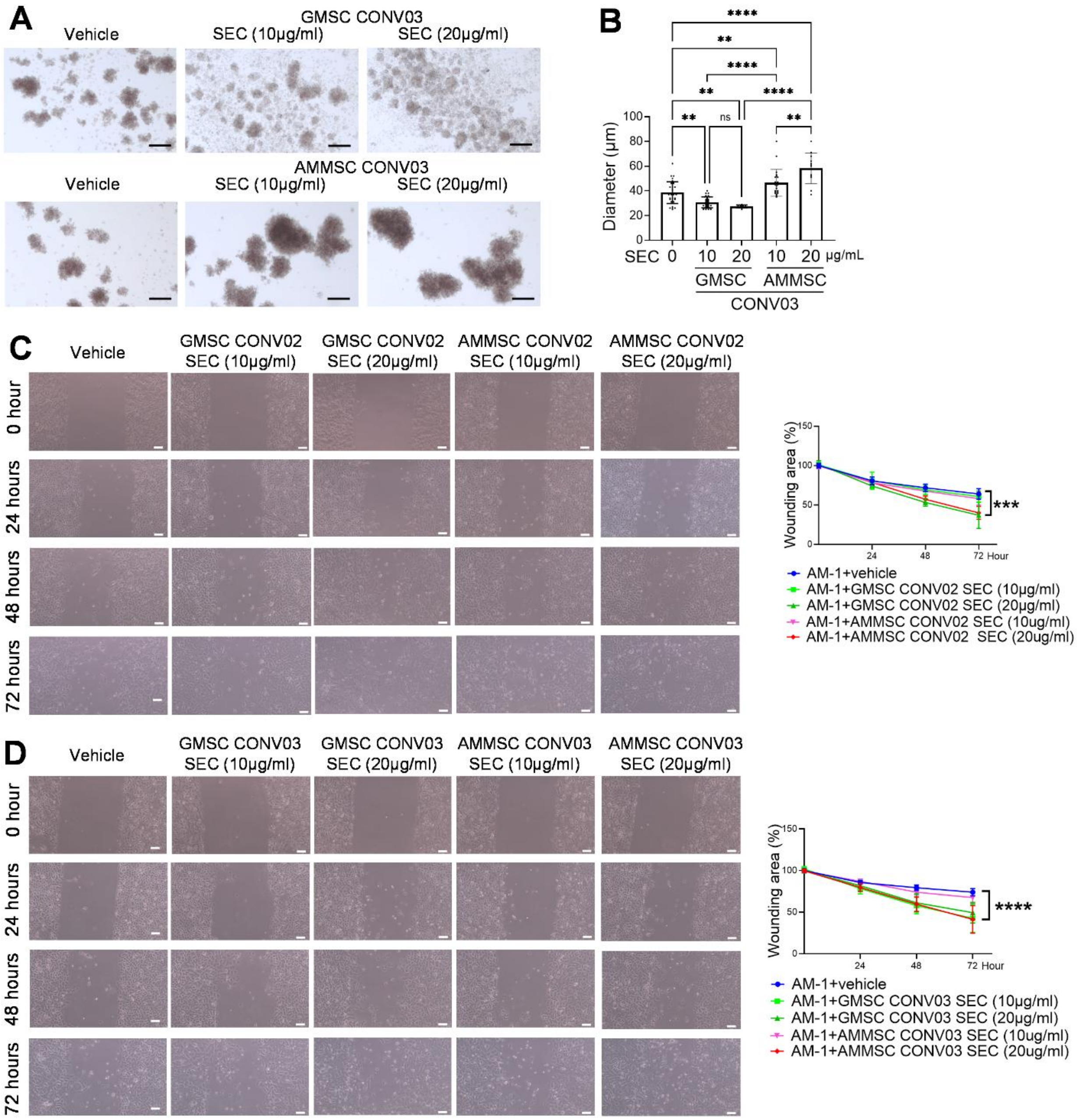

Figure S4. AMMSC-derived secretome promotes sphere formation and migration abilities. (A-B) Sphere formation of AM-1 cells treated with AMMSC CONV03-derived secretome (SEC) or GMSC CONV03 derived SEC for 10 days. Scale bars, 100 μm. Data are mean ± SD. One-way ANOVA, Tukey's multiple comparisons test. n=3 independent experiments from CONV03. ns= not significant, ***p*<0.01, *****p*<0.0001. (C-D) Wound healing assay of migrating AM-1 cells treated with GMSC CONV02/CONV03-derived secretome or AMMSC CONV02/CONV03-derived secretome. Scale bars, 30 μm. Data are mean ± SD. Two-way ANOVA, Tukey's multiple comparisons test. n=3 independent experiments from CONV02 and CONV03. ****p*<0.001, *****p*<0.0001. Related to Figure 1 and Figure 2.

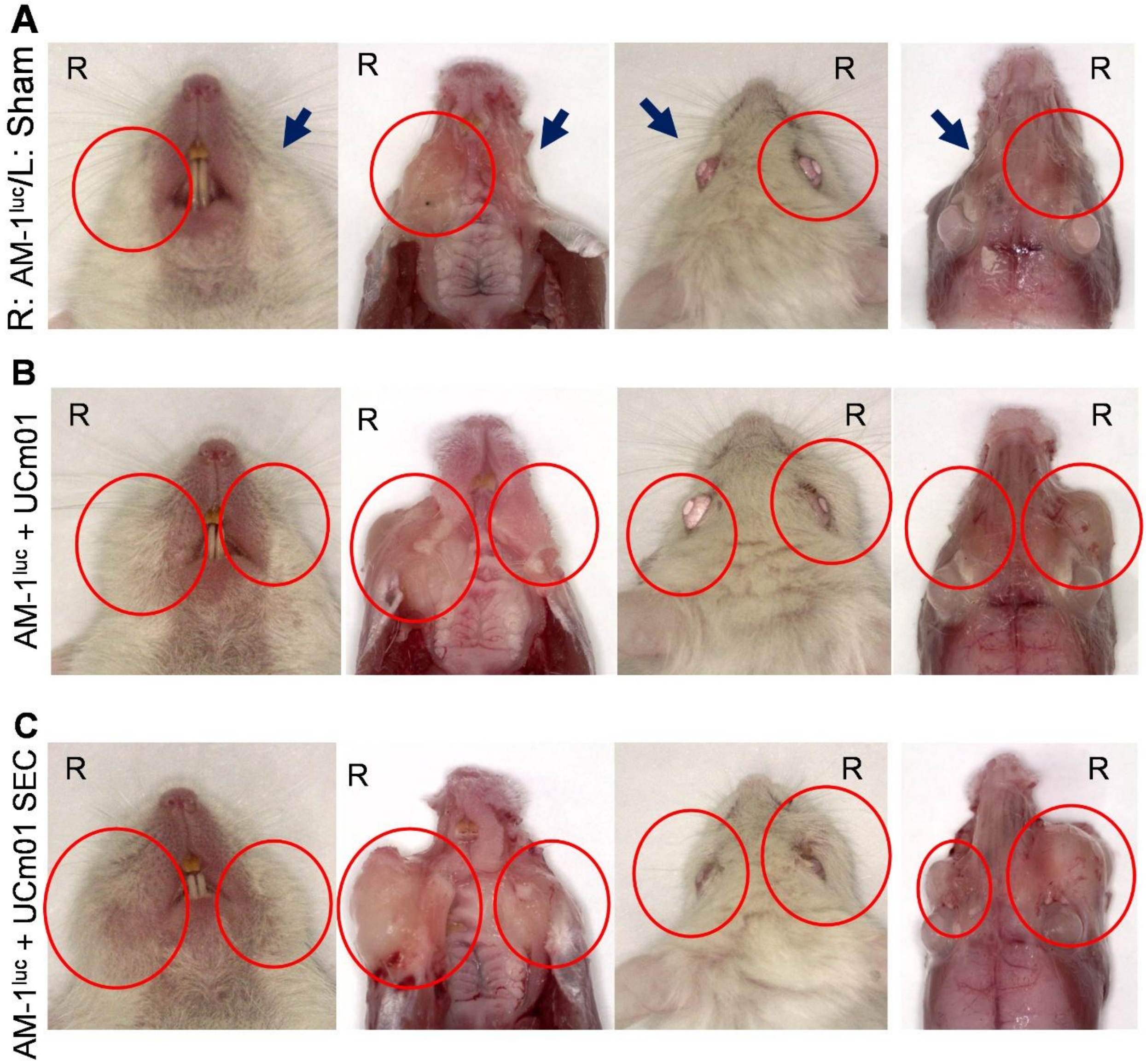

Figure S5. Bony expansion of orthotopic tumors in ASID mice. Representative images showing maxillary bony expansion (red circles) on Day90 after orthotopic transplantation of AM-1^{luc} cells alone (A), AM-1^{luc} cells co-transplanted with AMMSCs (B), or AM-1^{luc} cells pretreated with AMMSC-derived secretome (SEC) (C). In contrast, the sham control site exhibited a concave profile and a flat maxillary ridge (arrow) (A). Representative images are shown from four mice per group. Related to Figure 4.

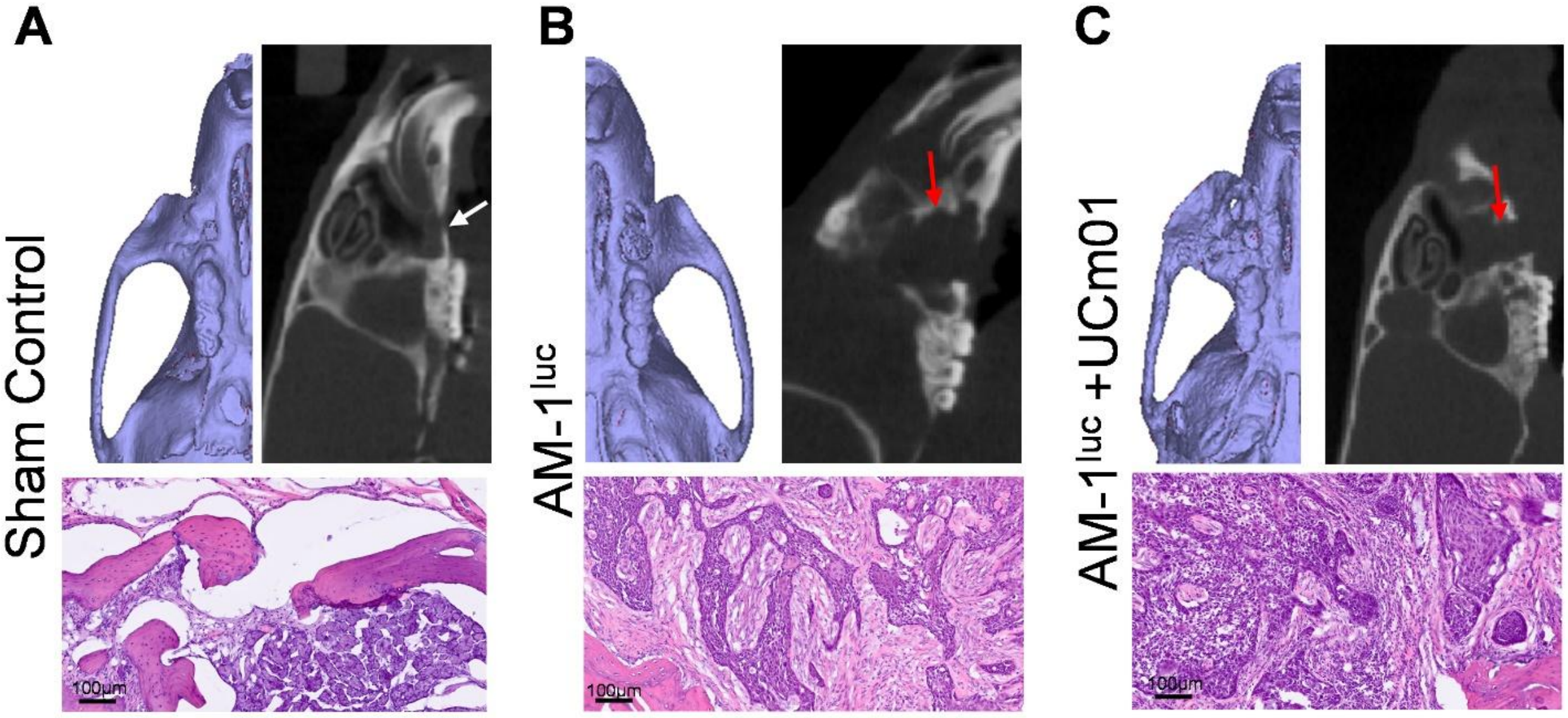

Figure S6. Intraosseous tumor formation in the maxilla of ASID mice. (A) Representative micro–computed tomography (micro-CT) image of the sham control site showing a healed maxillary ridge (white arrow) on Day 90 after surgery. (B and C) Representative micro-CT images showing multilocular radiolucent lesions (red arrows) on Day 90 after orthotopic transplantation of AM-1^{luc} cells alone (B) or AM-1^{luc} cells co-transplanted with AMMSCs (C). Corresponding H&E-stained sections demonstrate healed bone with fibrotic tissue in the sham control (A) and multiple epithelial tumor islands adjacent to bone in tumors formed by AM-1^{luc} cells alone (B) or AM-1^{luc} cells co-transplanted with AMMSCs (C). Scale bars, 100 μm. Representative images are shown from four mice per group. Related to Figure 4 and Figure 8.

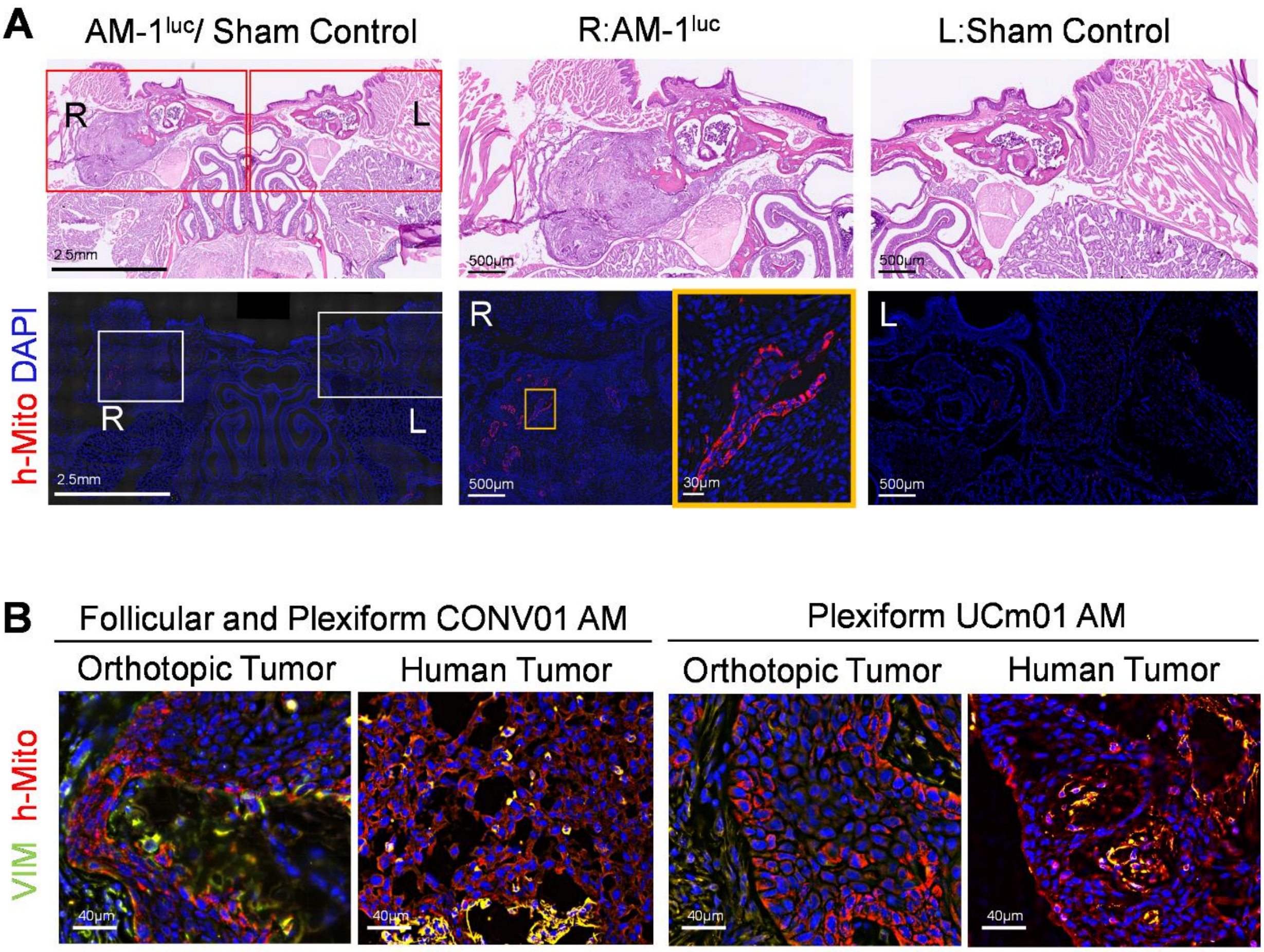

Figure S7. Localization of human mitochondria reveals reversed nuclear polarity in orthotopic ameloblastoma tumors. (A) Orthotopic tumors formed by AM-1^{luc} cells alone. Left: lower magnification of middle and left panels. Scale bars, 2.5 mm. Middle and left: scale bars, 500 µm. The central panel in lower middle panel: higher magnification of the yellow box. Scale bar, 30 µm. (B) Orthotopic tumors formed by AM-1^{luc} cells co-transplanted with AMMSCs, shown alongside corresponding human ameloblastoma tissues. Human mitochondria localization highlights the reversed nuclear polarity characteristic of ameloblastoma. Scale bars, 40 µm. All representative images from three independent experiments. Related to Figure 4.

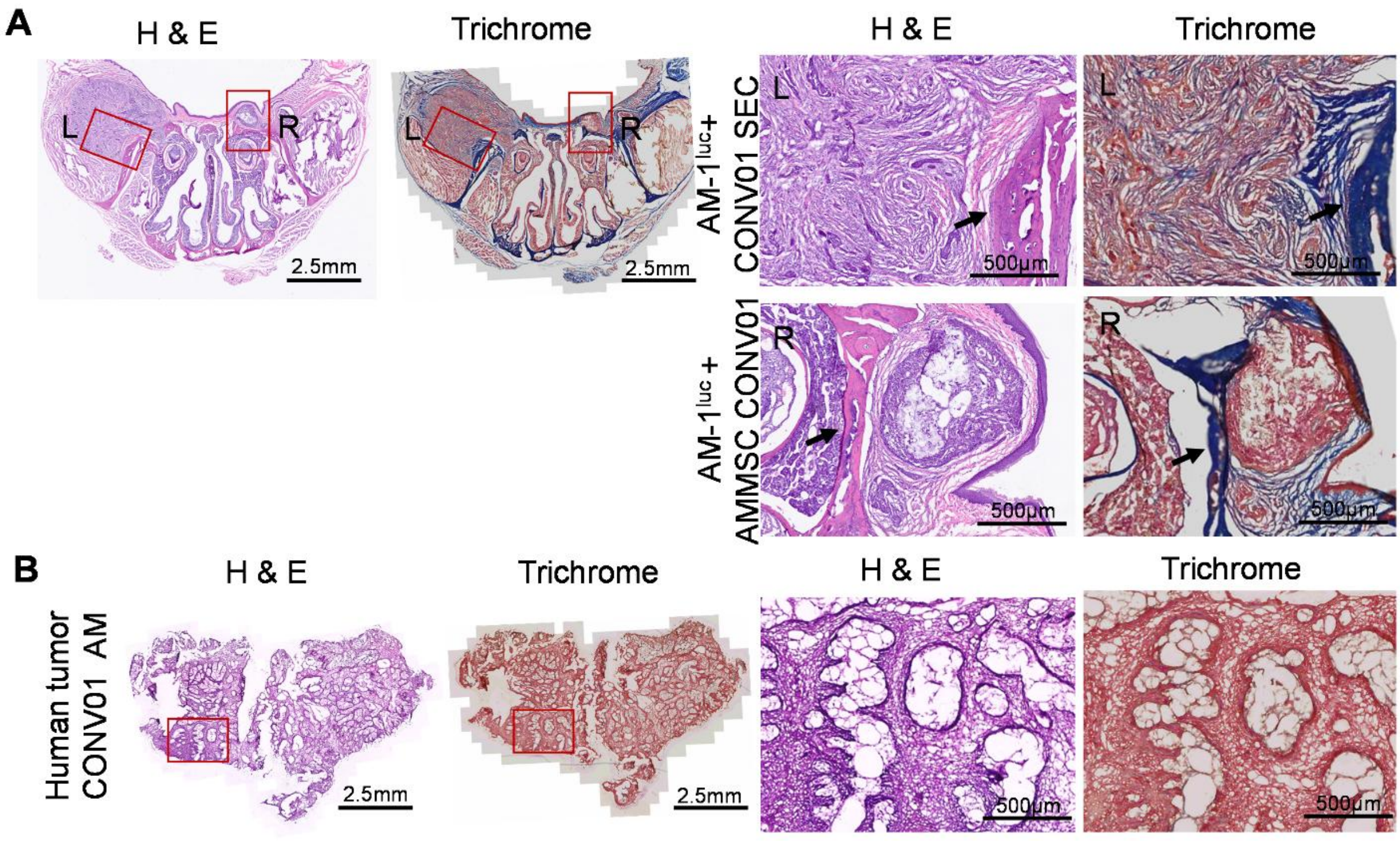

Figure S8. AMMSCs facilitate reconstruction of the ameloblastoma microenvironment in the orthotopic mouse model. Masson's trichrome staining reveals collagen deposition within the tumor microenvironment. (A) Orthotopic tumors formed by AM-1^{luc} cells co-transplanted with AMMSC CONV01 or pretreated with CONV01-derived secretome (SEC). Left two panels: lower magnification of right four panels. Scale bars, 2.5 mm. Right four panels: scale bars, 500 µm. (B) Corresponding human ameloblastoma tissue from CONV01. Left two panels: lower magnification of right four panels. Scale bars, 2.5 mm. Right two panels: scale bars, 500 µm. Representative images of two independent experiments. Related to Figure 5.

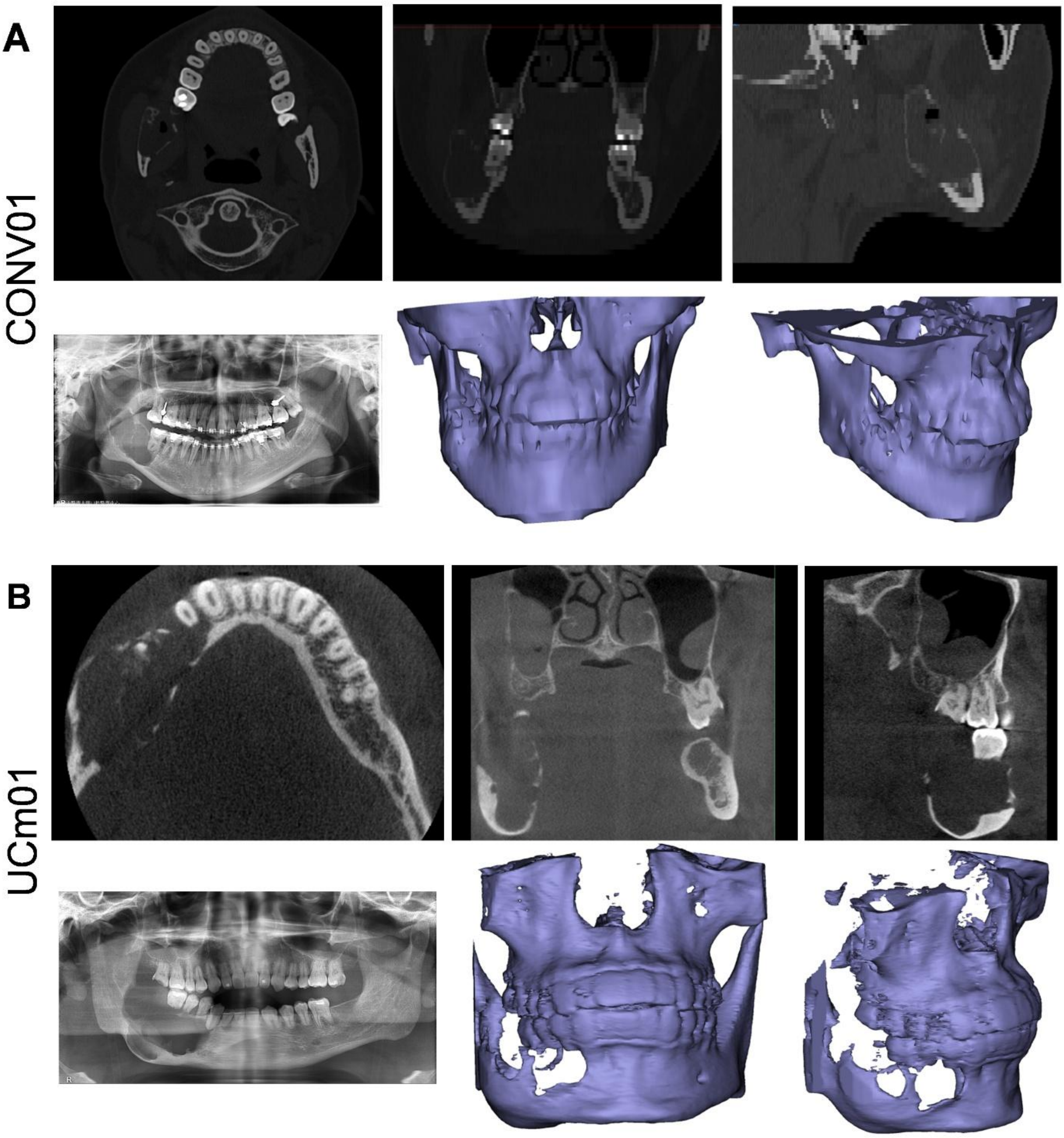

Figure S9. Clinical computed tomography and panoramic imaging of human ameloblastoma. (A) Representative clinical images from the ameloblastoma case CONV01. (B) Representative clinical images from the ameloblastoma case UCm01. For each case, the upper panels show axial (left), coronal (middle), and sagittal (right) views from computed tomography (CT). The lower panels show the panoramic radiograph (left) and three-dimensional CT reconstructions (middle and right). Related to Figure 8.

Patient ID	Sex	Age (years)	Ameloblastoma type	Histopathological subtype	Disease status	Derived primary cell culture(s)
CONV01	Female	22	Conventional	Follicular and plexiform	Primary	AMMSC CONV01
CONV02	Female	29	Conventional	Desmoplastic	Primary	AMMSC CONV02<br>AMEpiC CONV02
CONV03	Female	63	Conventional	Follicular and plexiform	Primary	AMMSC CONV03
CONV04	Male	59	Conventional	Follicular and plexiform	Primary	AMMSC CONV04
UCm01	Male	24	Unicystic, mural	Plexiform	Primary	AMMSC UCm01
UCm02	Male	45	Unicystic, mural	Plexiform	Recurrent	AMMSC UCm02

Table S1. Clinical characteristics of patients with ameloblastoma and corresponding primary cell cultures. Related to Figures 1-8 and Figures S1-S9.

Donor ID	Sex	Age (years)	Tissue source	Donor category	Derived primary cell culture(s)
CONV01	Female	22	Matched normal gingiva	Ameloblastoma patient	GMSC CONV01
CONV02	Female	29	Matched normal gingiva	Ameloblastoma patient	GMSC CONV02
CONV03	Female	63	Matched normal gingiva	Ameloblastoma patient	GMSC CONV03
UCm01	Male	24	Matched normal gingiva	Ameloblastoma patient	GMSC UCm01
GMSC01	Female	21	Gingiva	Healthy donor	GMSC01
GMSC02	Male	50	Gingiva	Healthy donor	GMSC02
18F	Female	18	Dental pulp (DP)	Healthy donor	DPMSC 18F
18F	Female	18	Dental follicle (DF)	Healthy donor	DFMSC 18F

Table S2. Characteristics of normal-tissue donors and corresponding primary cell cultures. Related to Figure 2 and Figures S3-S4.
